# A Comprehensive Survey of Driving Monitoring and Assistance Systems

**DOI:** 10.3390/s19112574

**Published:** 2019-06-06

**Authors:** Muhammad Qasim Khan, Sukhan Lee

**Affiliations:** Department of Electrical and Computer Engineering, Intelligent Systems Research Institute, Sungkyunkwan University, Suwon 440-746, Korea; qasim@skku.edu

**Keywords:** advanced driving assistance systems, aggressive and gentle driving, collision avoidance, distraction detection, fatigue detection, driving style recognition, vehicle detection and tracking

## Abstract

Improving a vehicle driver’s performance decreases the damage caused by, and chances of, road accidents. In recent decades, engineers and researchers have proposed several strategies to model and improve driving monitoring and assistance systems (DMAS). This work presents a comprehensive survey of the literature related to driving processes, the main reasons for road accidents, the methods of their early detection, and state-of-the-art strategies developed to assist drivers for a safe and comfortable driving experience. The studies focused on the three main elements of the driving process, viz. driver, vehicle, and driving environment are analytically reviewed in this work, and a comprehensive framework of DMAS, major research areas, and their interaction is explored. A well-designed DMAS improves the driving experience by continuously monitoring the critical parameters associated with the driver, vehicle, and surroundings by acquiring and processing the data obtained from multiple sensors. A discussion on the challenges associated with the current and future DMAS and their potential solutions is also presented.

## 1. Introduction

### 1.1. Background and Motivation

Transportation plays a vital role in individual and social welfare, the economy, and quality of life. Its benefits, however, are not a free lunch. Society pays in terms of money (for vehicles’ purchase, operational, and maintenance costs), social and ecological costs (resource utilization, exhaust and noise pollution, traffic jams), fatal or harmful traffic accidents, and so on. There are several measures to improve the quality of the modern transportation system at each level of society ranging from government policies to individual drivers’ performance [1]. A major objective of such improvements is called Vision Zero, which envisions a future where no one is seriously injured or killed in a road accident [2]. Vision Zero has a broader spectrum, however, this work will concentrate on the studies and systems developed to enhance road safety and driver performance. A significant portion of road accidents is attributed to drivers’ inattention and aggressive behavior [3,4]. According to reports [5,6,7] of the World Health Organization (WHO), every year approximately 1–1.24 million people are killed while 20–50 million people are injured on the roads across the world. Moreover, if the current tendency lasts for a decade, an increased rate as high as 60–70% of road accidents could make it the 5th main cause of death by 2030. In monetary terms, the costs involved in road accident damages are estimated at more than half a trillion USD, which makes nearly 2% of the gross national product (GNP) of advanced economies, 1.5% of GNP of medium income countries, and 1% of GNP of low-income countries.

In addition to driving safety, another emerging concept in vehicular technology is the comfort of the drivers. A driver’s mental and physical stress are studied in several studies (e.g., [8,9,10,11]) with the aim of providing a relaxed and comfortable driving experience. To achieve the target of safety and comfort, understanding and modeling of the driving environment which includes vehicle, driver, and surroundings has become a popular topic in multiple research areas. In fact, the topic is not confined in a specific field, but it overlaps among neuroscience, psychology, behavior science, signal and image processing, automotive engineering, artificial intelligence, control theory, and so on. During the last three decades, various comprehensive models are proposed to describe how these multiple research areas interact with each other. It is notable that driving safety and comfort are both important subjects and, despite inconsequential differences, the two topics cannot be entirely separated.

The objective of driving monitoring and assistance systems (DMAS) is to keep an eye on the driving status of a driver and to provide necessary assistance for safe and comfortable driving. Such systems assist drivers by easing their control efforts, reinforcing their sensing power, warning them in case of mistake, and so on. Depending on their functionalities, there are various names for such automation systems such as intelligent vehicle control systems, advanced driver assistance systems, collision avoidance systems, driver’s inattention monitoring systems, and so on. This work considers all such system as DMAS. Typically, driving assistance and monitoring systems go side by side and are mostly considered under the same concept. The subtle difference between the two can be realized as the driving monitoring system understands the driving situation, and assistance systems assist the drivers to handle the situation. Alternatively, the monitoring systems are more focused on safety while assistance systems have more to do with the drivers’ comfort [12,13]. Moreover, the terminology used for vehicles equipped with such systems is also diverse (e.g., intelligent vehicles, cognitive vehicles, and smart vehicles). Please note that this work does not concentrate on driverless or fully autonomous vehicles, though several ideas developed under the umbrella of DMAS serve as the foundation stone for fully autonomous vehicles.

### 1.2. Contribution and Organization

This work provides a comprehensive survey of DMAS taking into account the three main elements of the driving process (i.e., driver, vehicle, and driving environment). Intuitively, the topic is so wide and deep that no single paper can provide an in-depth analysis of all the research work being conducted in this field. This work, however, intends to benefit the researchers and interested readers by providing a comprehensive framework about the basic understanding of DMAS, major research areas and their interaction, and challenges arising in this field. For the interested readers, other survey papers and references provided in this paper can serve as additional resources on specific topics or on the areas remained unreported in this work. This paper is organized as follows:

- Basic concepts related to driving process and problems associated with a driver’s distraction, fatigue, and driving style are discussed in Section 2.
- Section 3 provides a survey of the driver-focused studies and explains the systems and techniques developed to detect a driver’s distraction and fatigue.
- A discussion on modeling and recognition of driving style behavior is provided in Section 4. These studies are mainly vehicle-oriented as the data used for driving style recognition is extracted from sensors installed on the vehicle.
- In Section 5, a review is presented on the models and systems developed to avoid collision by detecting other vehicles. Therefore, this section focuses on the driving environment.
- A review of systems developed to enhance a driver’s perception of comfortable driving experiences and DMAS available in modern vehicles is presented in Section 6. Moreover, a brief description of future trends in DMAS is also provided in the same section.
- Section 7 concludes this survey.

## 2. Basic Topics Associated with Driving

### 2.1. Driving Process

**Figure 1:**
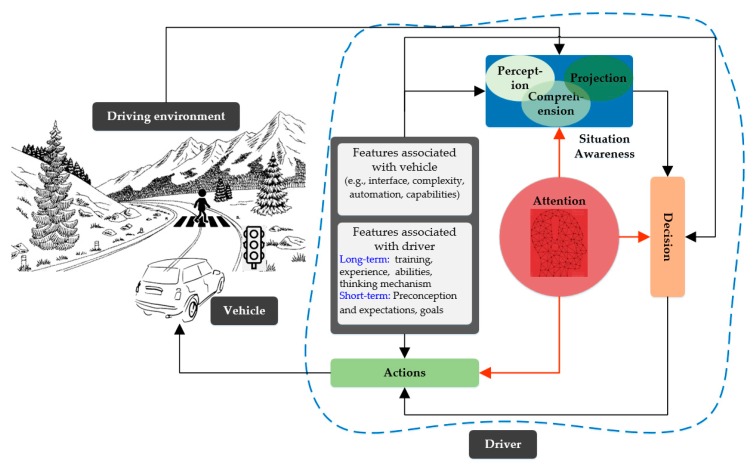
Driving process.

Driving is a dynamic process whose key elements are driver, vehicle, and driving environment (e.g., traffic, road signs, and pedestrians) as shown in Figure 1. The primary function of a driver is to remain aware of the environment, make decisions, and perform the actions [14]. In Figure 1, these stages of the driving process are shown where situation awareness is considered to be the most complicated stage. In [14], situation awareness is modeled as a three-level process consisting of perception of the elements in the environment within a volume of time and space, comprehension of their significance, and projection of their impact in the near future. A driver’s ability to accurately perceive multiple items in parallel requires attention in the perception phase, and situation awareness mainly depends on it. In addition to its application in the later stages of Decision and Actions, attention is necessary to take in and process the available indications. The importance of a driver’s active attention increases in a vibrant and complex driving environment for the sake of life and property safety. That is why a continuous monitoring of the driver’s attention is a primary concern for safe driving and has been an active research area for decades. To ensure driving safety after the inattentive behavior of a driver is identified, various countermeasures are adopted depending on the nature and intensity of the inattention. The main reasons for road accidents that account for more than 90% of total accidents are summarized as [15]:

1. Distraction (ranging from mild distraction to, looked but could not see, status which is a form of cognitive distraction)
2. Fatigue (this work considers it as a comprehensive term which also encompasses the drowsy behavior of a driver)
3. Aggressive driving style (which is typically detected by vehicle-related parameters such as sharp turns, over-speed, and hard braking)

### 2.2. Distraction

Driving itself is a serious job. A driver’s engagement in a cognitively demanding parallel task impacts the driver’s performance. It is reported that distraction is the major cause of road accidents which accounts for more than half of the total accidents [4,16]. The common types of distracting activities include eating or drinking, looking at off-road persons and events, operating in-vehicle-technology, texting, and listening to a phone [15,17,18]. The major categories of distraction are summarized as follows with the first two categories being at the early stages of the research [3,4,19]:

- Olfactory distraction
- Gustatory distraction
- Visual distraction
- Auditory distraction
- Biomechanical distraction
- Cognitive distraction

Several metrics, such as gaze patterns and head movements, are proposed and studied in the literature to detect a driver’s distraction [20,21]. The pattern of looking ahead changes when a driver is involved in a cognitively demanding task other than driving. The studies in [22,23] show that an analysis of gaze pattern can discriminate between focused and distracted driving, and provides a relative measure of cognitive involvement (i.e., either task is easy or difficult). In [24,25], it is reported that distracted drivers mostly keep looking directly ahead and have lesser glances at traffic signals and area around the intersections. Moreover, the study in [25] reported shrinkage of the visual field by 13.6% and 7.8% during a counting task of high and average difficulty levels, respectively. A reduction in saccades per minute indicates a reduced exploration of driving surroundings (including complete unawareness of some areas and tasks at times) and lower glance frequency for distracted drivers [26]. It is shown that distraction, cognitive workload, and features of eye movement (saccade, smooth pursuit, and fixation) are interlinked [27]. Saccades are quick actions that occur when visual attention transfers from one point to another. Smooth pursuit occurs when a viewer visually follows a traveling object. Fixation happens when a spectator’s eyes are almost stationary. Since saccade distance decreases with an increase of task complexity, saccade is used as a helpful index for mental workload measurements [28].

On the other hand, head movements increase as the cognitive workload of a driver increases. It is reasoned that the increased head movements are compensatory actions of the driver to acquire a broader field of view [29]. According to [29], the status of the cognitive distraction of drivers can be appropriately detected with the help of standard deviation of head and eye movements. The study in [30] reveals that—frequent glances at an object far way and off-road results in increased visual distraction; blink frequency increases with cognitive distraction; and, the concentration of gaze and reduced rate of saccades are symbols of visual and cognitive distraction for a driver. As reported in [31], three parameters, viz. standard deviation of lane position, glance duration of head-off-road, and eyes-off-road glance time are crucial indicators of visual distractions.

In addition to eye and head movements, other physiological parameters also indicate a driver’s distraction. The authors of [32] discovered a reproducible effect that a driver’s engagement in a secondary cognitively demanding work such as talking to another person in the vehicle decreases the temperature at the tip of the driver’s nose. A consistent increase in skin temperature at the supraorbital region during visual and cognitive distraction is reported in [33]. The authors of [34] observed that EEG signals contain information about the mental workload and level of task engagement (to be explained later).

A driver’s distraction degrades the driving performance, and problems such as unplanned speed changes, hiccups in vehicle control, and drifting outside the lane edges are associated with distraction [35]. The author of [36] studied the lane-changing behavior of distracted drivers. The results reveal an increased delay and reduced frequency of circumspect approach (i.e., checking speed and mirrors, providing indicators at turns, etc.). Moreover, the effects of visual distraction are not the same as that of cognitive distraction. Visual distraction disturbs lateral vehicle control and steering ability of a driver, whereas longitudinal vehicle control is affected by cognitive distraction [37]. Similarly, the study in [30] reveals that visual distraction is associated with overcompensation and steering neglect, while under-compensation has a relationship with cognitive distraction. The authors of [24,26] suggest that hard braking is mainly related to cognitive distraction rather than visual distraction. An apparent inconsistent result reported in [37] mentions of a driver’s enhanced lateral control ability while performing a parallel cognitively demanding task. Such observations, however, need further research with an increased number of participants. Generally, the adverse effects of visual distraction are more than that of cognitive distraction.

### 2.3. Fatigue

Fatigue denotes a combination of symptoms like a subjective feeling of drowsiness and compromised performance, and its concept is different from that of distraction. The European Transport Safety Council (ETSC) states that fatigue “concerns the inability or disinclination to continue an activity” [38]. It is notable that despite the considerable research in this field, the term fatigue still has variation in its definition [39]. Thus, it is not straight-forward to ascertain the percentage of accidents related to fatigue. However, several studies reveal that 25–35% of driving mishaps are related to fatigue [40], making it the second major reason for road accidents. According to [41], at least one out of three drivers admitted that he/she fell asleep or nodded off at least once in his/her driving career. A driver’s drowsiness is usually caused by mental and central nervous fatigues which are the most dangerous types of fatigue during driving. The other kinds of fatigue are general physical fatigue (such as felt after exhaustive manual work) and local physical fatigue (such as in a skeletal muscle).

A fatigued driver undergoes certain physiological and physical phenomena and variations in body activities. The researchers have used these symptoms to detect the fatigued behavior of a driver. It is reported that a fatigued driver exhibits a deteriorated performance in steering-wheel control [42], a decreased rate of the steering-wheel reversal [43], driving without steering tweaking for an extended time span and a jerky motion afterward [44], movements of the steering wheel are of high amplitude with a larger standard deviation of angle [45], and low-velocity of steering wheel movements [46]. It is also discovered that a fatigued driver demonstrates an irregular pattern of vehicle tracking and angular movement of the steering wheel with a significantly increased range of deviation [47]. As the time of a specific task increases, fatigue accumulates and ability to follow the lane decreases [42]. Similarly, maximum lane deviation and count of lane deviations are highly correlated with eye closure rate and fatigue [48]. The quantitative measures such as standard deviation of lateral position and mean square of lane deviation are highly disturbed for a driver in fatigued status, and such measures serve as fatigue indicators [49]. Similarly, a study in [46] revealed that mean deviation in yaw position calculated over a period of 3-min and variance of yaw deviation is affected by driver’s drowsiness, and these two measures also make a good indicator for fatigue detection. The effects of fatigue on other parameters except steering-wheel control are not as prominent. Some studies (e.g., [50]) mention that after the third hour of driving, the standard deviation of speed increases with a 45 min time interval. However, according to [46], there is no convincing correlation of fatigue with acceleration or brake as the vehicle speed changeability depends on several other factors and has no strong correlation with fatigue [51].

An important indicator of fatigue detection known as PERCLOS is associated with the pattern of eye-blinking which measures the percentage of time when the eye is more than 80% closed [52,53]. Validated through subjective measures and EEG results, PERCLOSE is among the widely accepted criteria in the field of sleep research [54]. In addition to eye-related activities, other biological and physiological activities and parameters associated with driver’s fatigue such as electroencephalography are also utilized in DMAS. A detailed discussion of such parameters is provided later.

Fatigue has appalling effects on driving performance, and behavior of different drivers differs regarding various driving performance parameters. A summary of symptoms associated with fatigue, as discussed above and observed in other studies (e.g., [55,56,57]) is provided as:

- Frequent yawning
- Radically increased eye-blinking frequency
- Burning feeling in the eyes and hard to keep them open
- Lethargic or relaxed position of hands on steering wheel
- Increased (or sometimes irrationally decreased) response time
- Vehicle wandering between the lanes or out of road
- Nodding off and prompting the body or head from nodding off
- Shallow breathing
- A spontaneous head nod after glancing at side mirrors
- Reduced movement of the head
- Increased frequency of scratching legs, chin, head, and ears
- Turning head to the left to relieve the muscular tension of the neck
- Feelings of depression and irritation

It is intuitive that different drivers must have varying symptoms with a range of variations. Thus, there is no specific technique to gauge the fatigue level. Moreover, it is reported that numerous factors affect the physiological waking capacity and raise/lower the fatigue threshold. These factors (such as taking a shower before driving raises the fatigue threshold, and disturbed sleep or heavy labor lower this value) expedite or delay the appearance of fatigue effects on the driving process [38,39,40,58].

### 2.4. Driving Style

In addition to distraction and fatigue, another main reason for road accidents is the aggressive driving behavior which is usually observed in the form of ignoring the traffic signals and shortcut maneuvers. Such practices are predominantly serious to pedestrians, bicyclists, and motorcyclists who usually do not have much protection [59,60]. It is reported that accident rate due to aggressive or immature driving is higher in Asian countries compared to other countries [59]. The usual forms of improper driving behavior include:

- Ignoring speed limits and road conditions
- Opposite side driving
- Driving between two lanes
- Not using the indicator while taking a turn
- Driving when the driver is under the effect of drugs

It is observed that an alert and healthy driver whose physical and physiological parameters are within the normal ranges may exhibit such aggressive behaviors. Thus, such practices are commonly detected by vehicle-related parameters such as over-speeding or sharp turns. Therefore, in this work, the objective of driving style recognition is considered under vehicle-related studies rather driver-related studies. 

**Figure 2:**
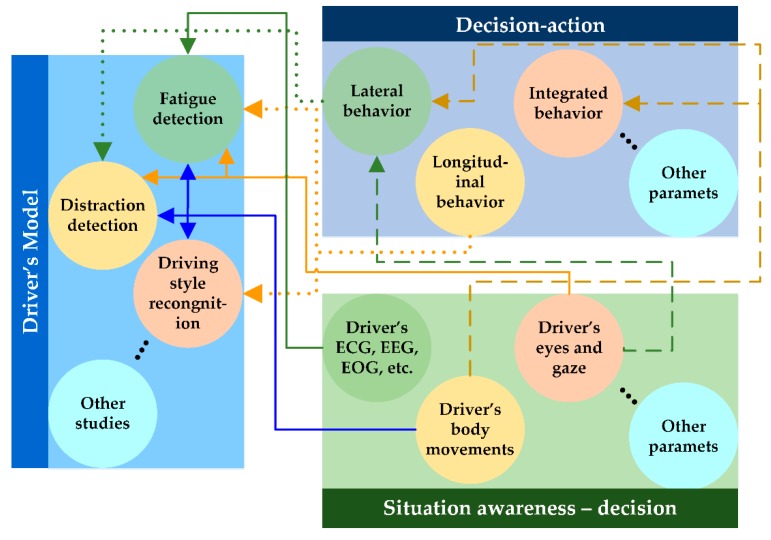
Interconnection of research areas.

Figure 2 provides a comprehensive outlook of this survey paper. With reference to Figure 1, the studies related to situation awareness-decision emphasize on how a decision is made in specific situation, whereas execution of the decision is investigated under decision-action related studies. The (solid, dotted, and dashed) lines show how the research areas involved in DMAS are interconnected. For example, a fixed gaze or closed eyes for a long period often indicates fatigue and a distracted gaze is a result of distraction (solid yellow line). Poor steering performance such as larger overshoot indicates how distraction affects the lateral driving behavior (green dotted line). Moreover, it is observed that the research areas involved in DMAS are, in general, indiscernible (e.g., an impulsive arm movement may belong to a sleepy driver (fatigue detection) or maybe an indicator of aggressive driving (driving style recognition)).

## 3. Driver-Focused Studies and Systems

**Figure 3:**
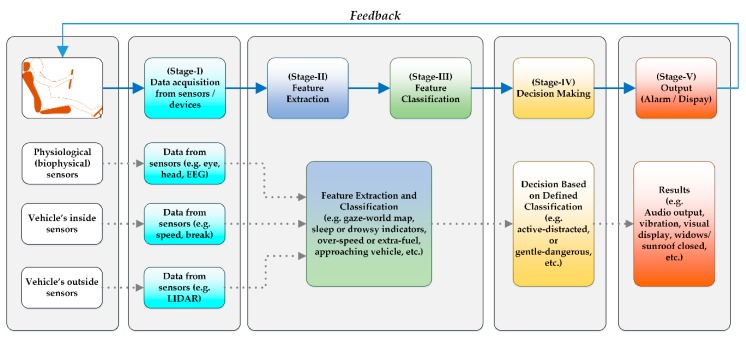
Layout of a typical DMAS.

A driver’s attentiveness plays the main role in safe driving. Figure 3 shows the layout of a typical DMAS designed to improve the driver’s attentiveness. It continuously monitors the parameters associated with the driver, vehicle, and surroundings by acquiring data from multiple sensors associated with: (a). The driver’s body, (b) installed inside, and (c) outer side of the vehicle. The acquired data is then processed to extract the required features based on which decision is made, and conveyed to the driver as shown in Figure 3. In this section, a survey of those studies and systems is presented that are related to the driver’s biological and physiological information. This information is acquired from the human body through intrusive and non-intrusive electrodes and provides decisive clues about the driver’s attentiveness.

### 3.1. Electroencephalogram (EEG)

EEG has a temporal resolution of 0.001 s and a spatial resolution of 20 mm, and is extensively used in the field of brain activity research. Using the frequency-domain features of EEG data (e.g., mean frequency, center of gravity of the EEG spectrum, and energy contents of α, β, θ, δ bands), a driver’s fatigue can be efficiently detected. Similarly, the time-domain features of EEG data, such as standard deviation, average value, and the sum of amplitudes’ squares, provide valuable information related to brain activity. In addition to differentiating between awake and asleep stages, an EEG is also employed to discern the different sleep stages.

Processing the data derived from the four EEG activities (α, β, θ, and δ), the authors of [61] proposed a five-level objective sleepiness score criteria which is often used as a validating standard for drowsiness detection algorithms. In most of the recent studies, the classification of a driver’s drowsiness state is carried out through modern classification techniques. The authors of [62] predicted a transition from vigilance to sleepiness by training of support vector machine (SVM) to classify EEG signals into four major frequency bands. For distinguishing mental fatigue into various levels, a comparison between a standard multiclass SVM and a probabilistic-based SVM is presented [63] which shows a better performance of the probabilistic-based multiclass SVM. The study in [64] developed a linear regression model to assess the sleepiness level. The deployment of this model on 33-channel EEG signals from the independent component analysis showed an accuracy of 87%.

In addition to fatigue detection, EEG data is also used to estimate the distraction level. In several experiments (e.g., [65,66]) involving those which required a second-by-second data [34], an association between EEG data and mental engagement levels is revealed.

The main limitation of EEG-based drowsiness detection systems is associated with EEG data collection for which the electrodes are placed on the head. Due to its complex arrangement and influence on the driver’s performance, this arrangement is not practical for real life driving. However, other alternatives, such as in-ear EEG electrodes, are also available in the market [67].

### 3.2. Electrocardiogram (ECG)

The ECG produces a graph of the electrical activity of the heart on a voltage versus time scale. The information collected through ECG signals such as heart rate, heart rate variability, and respiration rate provides valuable information related to driver’s fatigue as explained below:

1. Heart Rate (HR): A reduction in HR or the number of heart beats per minute is reported in [68,69] for persons moving from attentive to a sleepy state. Similarly, a decrease in HR is reported during long drives at night [50]. Moreover, a driver’s emotions, mental activity, and body exertion also affect HR [70,71].
2. Heart Rate Variability (HRV): HRV is the change in the time interval between two successive heart beats, and is also known as RRI. The activities of the autonomous nervous system (ANS) change due to fatigue or stress and can be efficiently detected by HRV [72,73,74]. The studies show that a reduction in HRV is observed as workload increases, indicating a negative correlation between HRV and workload. It is notable that certain activities are mentally easy and physically hard, whereas several activities are mentally hard and physically easy. Typically, during the second type of activities, HR increases and HRV decreases [72,75]. Apart from the HRV pattern, the power spectral analysis of HRV also provides valuable information for drowsiness detection [69]. According to studies on ECG data, HRV receives the priority for early fatigue detection. The instantaneous deviation observed in time-domain ECG signal is the main drawback of HRV [76], which is resolved by its time-frequency analysis [77].
3. Respiration Rate (RR): RR is the count of breaths exhaled and inhaled in one minute. The authors of [78] tried to establish a link between drowsiness and RR according to which RR starts to fall with the initialization of drowsiness and sets in, and continues to fall until sleep onset. However, this observation did not receive consensus. For example, the study in [76] experimented 34 participants but did not find any significant variations in the respiratory cycle due to sleepiness. It is observed that non-contact ECG measurements require a close proximity to the driver; otherwise the accuracy of results is compromised [78].

### 3.3. Electrooculography (EOG)

Used for recording eye movements, EOG provides a measure of the corneo-retinal standing potential between the front and the back of the human eye which typically ranges from 0.05–3.5 mV [79]. The resulting signal is called the electrooculogram. Eye activities, such as eye movement and blinking, change this potential difference and result in a variation of the EOG signal [61,80]. For example, a blink is detected when the contact between the eye’s upper and lower lids lasts for about 200–400 ms, and a microsleep is detected if the eye remains closed for more than 500 ms [61,81].

The drowsiness of a driver can either be detected by (i) eyelid movement-based indicators (such as amplitude, duration, and frequency of blinking); and (ii) eyeball movement-based indicators (such as slow eye movement and rapid eye movement). A brief description of the most commonly used eye-related parameters used in the field of drowsiness research is presented as follows [79,81,82,83,84]:

- Blink Duration: Blink duration is a measure of the total time (ms) from the start to the end of a blink.
- Blink Frequency: It is the number of blinks in a minute. An increased blink frequency is an indicator of drowsiness.
- Blink Amplitude: It provides the measure of electrical potential during a blink. Blink amplitude is measured by EOG electrodes and its typical range is 100–400 μV.
- PERCLOS: The proportion of time during which the eyes remain at least 80% closed in one minute.
- Lid Reopening Delay: The time taken from full closure of lid to the start of its reopening. Its duration is a few milliseconds for an awake person, and it increases during drowsiness and extends to several hundred milliseconds during a microsleep.
- Eye Ball Movement: The eyeball movements take place when eyeball moves from its point of fixation. This phenomenon is also used as an indicator of drowsiness.

The placement of EOG electrodes assumes particular importance when collecting EOG data. The farther the electrodes from the eyes, the more vulnerable is the accuracy of the collected EOG signal [85,86]. Moreover, attaching electrodes near the eyes is disturbing for drivers. Further, drowsiness detection schemes based on blink behavior are strongly person-dependent. For mentally retarded persons, such schemes may not work well as they may perform more numbers of blinks in wakeful conditions or their eyes may remain open even in drowsy conditions [84].

### 3.4. Electromyography (EMG)

EMG is a technique for evaluating and recording the electrical signal generated from muscle contraction [87,88,89]. The studies reveal that there is a link between EMG amplitude and muscle fatigue as the amplitude of EMG signals decreases gradually with fatigue. The analysis of EMG data provided in [90,91,92,93,94] establishes a correlation between muscular fatigue and drowsiness. During muscle contraction, a shift in center frequency component is observed towards the lower spectral band [95,96].

The major drawback of the EMG signal lies with its random and complex nature and dependency on the biological and structural properties of the muscle [79,97].

### 3.5. Electro-Dermal Activity (EDA)

EDA, previously also known as galvanic skin response (GSR), provides a measure of skin conductance which changes due to the secretion of sweat gland. Sympathetic arousal of ANS controls the secretion of the sweat gland. During drowsiness, the activity of the parasympathetic nervous system is triggered which reduces sweating. Consequently, the resistivity of skin increases and skin conductivity decreases [93,98,99]. In this way, EDA provides a measure of drowsiness. However, this technique is highly sensitive to atmospheric humidity and temperature.

### 3.6. Skin Temperature (ST)

ST is maintained within a certain range by the thermoregulation system of the human body. The ST measurement methods measure the temperature of the skin surface which varies with the level of drowsiness. In [100], for example, five levels of drowsiness are described by measuring the temperature of nasal skin, forehead temperature, and tympanum temperature. The first two measurements are based on ST, whereas the last measurement represents the core temperature of the human body (i.e., the working temperature of the body organs).

### 3.7. Hybrid Techniques

The already discussed parameters have certain benefits and limitations in comparison to one another. So, trusting on a single parameter to detect drowsiness may lead to erratic results. Hence, to increase the accuracy of the detection system, several studies utilize a combination of multiple parameters for drowsiness detection. A few examples of such studies are—the work in [74] used a combination of data from breathing frequency and HRV; the work in [76] used a combination of data from RRI, HRV spectral power, RR, EEG band power, and EMG; the authors of [78] exploited HR, HRV, and blinking and breathing rate information; the study in [93] combined EDA and EMG data; the authors of [101] utilized EEG energy and band power, HRV spectral components, and sample entropy; the study in [102] utilized ECG entropy and EEG spectral power; the work in [103] combined the information obtained from PERCLOS and EEG band power.

A summary of studies related to the driver’s attention monitoring based on biological and physiological parameters is provided in Table 1.

## 4. Vehicle-Focused Studies and Systems

**Figure 4:**
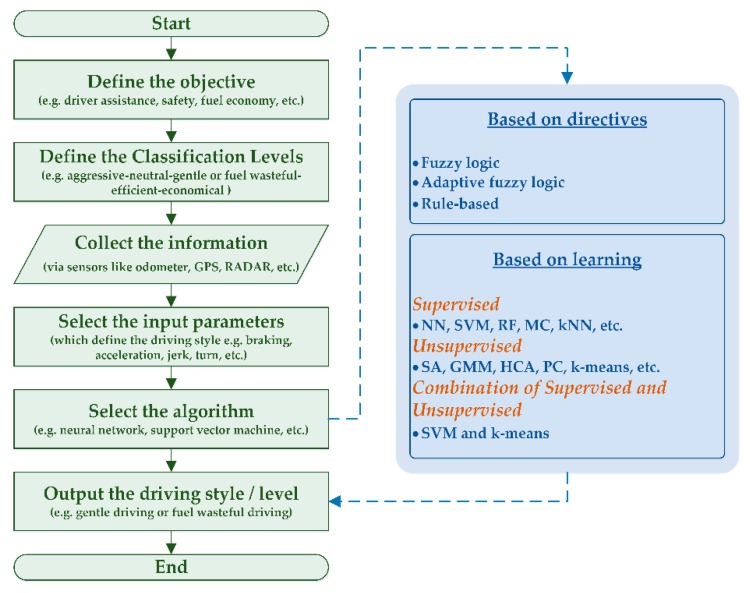
Design of a generic driving style recognition program.

Understanding the driving style through vehicle-associated parameters is an important topic of DMAS which helps in providing improved on-road safety, economic mobility, and a greener environment. Moreover, knowledge of driving style is also mandatory for the development of future DMAS and autonomous transportation systems [119,120,121]. Recognition of driving style is a complex multidisciplinary topic influenced by several environmental (e.g., weather, season, time of the day, and lighting condition) and human (e.g., age, gender, and behavior) factors. It is notable that DMAS that assists drivers during certain events are typically designed while considering average driver characteristics. Though the calibration accommodates for a wide range, yet, it cannot adapt to the precise singularities of a specific driver [122,123,124]. Hence, the future DMAS aims for driver style recognition to personalize the system performance, enhance safety, and improve the fuel economy [124,125]. A flowchart of a generic driving style recognition program is shown in Figure 4 as explained below.

### 4.1. Definition of the Objective

The first step in a typical driving style recognition model is to define the objectives of the program. In the majority of the models, the primary objective is based on safe driving behavior. Other objectives may include fuel economy and behavioral analysis as shown in Table 2.

### 4.2. Classification Levels

The next step is to define the number and type of classification levels. The driving style classification levels, which are also associated with classification criteria, recognition algorithm, and input signals, are broadly categorized into the following two categories:

1. Discrete Classes: Driving styles are often categorized into discrete classes on the basis of selected driving parameters and extracted features as shown in the initial rows of Table 2. These classes are defined at the design stage of the classification algorithm and encompass all values of input parameters to produce a multifactor classification. Titles or labels of the classes are based on the classification objective, such as safety or fuel economy. Applications related to safety define classes and assume title based on aggressive or gentle behavior of the driver, while fuel-related classification generally uses terminology such as efficient or economical. With the increased research in this field, it is expected that further classification criteria and labeling titles will increase.
2. Continuous Scale: Instead of discrete classes, this classification style takes into account a higher number of clusters through continuous indexing. To produce the output, it is possible to use a threshold-based algorithm that converts the continuous values into finite classes [136,144]. The classification approach of continuous indexing has been adopted in recognition of driving styles related to safety, behavioral analysis, and fuel economy as shown in Table 2. The work in [144] classifies driving style in a range of (−1, 1) whereas 0 represents a neutral driving style with gentle and aggressive styles at the corners. An aggressive driver tends to drive the vehicle in a risky manner, ignoring speed limits, improper car-following, changing lanes erratically, and hasty turns. Similarly, a driving style classification is developed in [141,142,143,145] based on vehicle efficiency calculated through fuel consumption.

### 4.3. Information Collection

The information is collected through instrumentation installed inside as well as outside the vehicle (e.g., inertial measurement units, differential GPS, and radar). As shown in Table 2, the data acquired about speed, fuel consumption, acceleration, throttle, braking power and frequency, throttle, jerk, sharp turn, and deceleration, helps in defining the driving style. Similarly, event related information, such as the speed at roundabouts, sharp turns, and car-following, is also used in classification algorithms. 

### 4.4. Selection of Input Variables

Another step for driving style recognition is to determine the variables required to be monitored for the selected classification algorithm. Identifying the correct signals is a key factor as further processing and results are based on it. However, no general agreement is found on the recommended set of signals for a certain task in the literature [140]. This disagreement results in the variety of driving style recognition models for the driver’s behavioral analysis, fuel economy, and safety enhancement as shown in Table 2.

### 4.5. Classification Algorithm

Development of the driving style recognition algorithms is based on the input signals and classification method and levels. These algorithms are usually based on machine learning techniques and methods based on directives (e.g., fuzzy logic, rule-based) as shown in Figure 4. Typically, a complete driving style recognition program incorporates a combination of various techniques. For example, the first data is processed through a machine learning technique, and then a rule-based classification is applied to produce the output result. Main categories of algorithms proposed in the literature are summarized as follows:

- Fuzzy logic (e.g., [125,134,146,147,148])
- Adaptive fuzzy logic (e.g., [129])
- Rule-based (e.g., [126,136,140,141,143,149])
- Supervised learning (e.g., [128,130,131,135,144])
- Unsupervised learning (e.g., [133,150,151])
- Combination of different techniques (e.g., [152])

## 5. Driving Environment-Focused Studies and Systems

**Figure 5:**
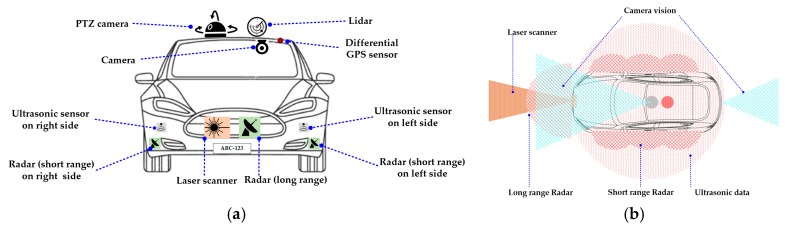
Sensors on a vehicle (**a**) typical location of sensors; (**b**) the working field of various sensors (the two pictures are for description purpose and do not correspond to one another).

In addition to observing a driver’s focus, DMAS also detect and track the surrounding vehicles and pedestrians to enhance the driver’s attentiveness and to avoid any possible collisions. Conventionally, various inert systems are available in the vehicles for decades that reduce the level of mutilation during and after a collision. Airbags, crumple zones, seatbelts, and laminated windshields are examples of such systems. However, in this section, a survey of those vigilant sensors and systems is provided which use vehicle detection and tracking technology to reduce the risk and damage of an accident. These systems provide the driver with information about vehicles in proximity, their gap, and relative velocities. This information is extracted with the help of passive and active sensors which acquire nearby traffic data, and then apply vehicle detection and tracking algorithms to this data. When an imminent collision is estimated, these systems warn the driver and/or prepare the necessary systems, such as brake and steering, for a safe exit. Figure 5 shows the typical installation place and field of work of these sensors. The sensors used for vehicle detection are broadly categorized into the following categories.

### 5.1. Passive Sensors

Passive sensors acquire data in a nonintrusive way as they receive signals without emitting them. These sensors work on the principles of sound and light detection. Examples of acoustic sensor and optical sensors are provided in [153] and [154,155], respectively. Based on the acoustic signal, a scheme for real-time detection and tracking of an approximating vehicle is proposed in [153]. In the first step, it employs a gradient method to extract the robust spatial features from noisy acoustical data. At the later stage, the acquired spatial features are processed through sequential state estimation to produce the output. The proposed scheme was verified with practical acoustic data. The work in [156] proposed a comprehensive design of an acoustic detection prototype hardware to sense nearby traffic by estimating road congestion using noise as a negative feature of the urban roads. After sampling the road noise, it is processed to compute important parameters such as vehicle speed. The speeds are estimated using honks data and implementing differential Doppler shifts. The acquired parameters were transmitted to a remote server every minute. The traffic condition on the road was determined by a remote processor using values of these parameters.

Optical sensors use single [157], multiple [158,159], or stereo cameras [160] to track the approaching and preceding vehicles. The cameras are mounted inside the vehicle near the back-view mirror and on the rear-side of the vehicle. In certain applications, more than one camera or pan-tilt-zoom (PTZ) cameras are required to capture a 180–360° view of the surroundings. Due to the poor performance of normal cameras under low light conditions, infrared (IR) cameras are utilized for night-time applications [154]. Both monocular and stereo vision signals are utilized for vehicle detection and tracking, with the typical application of stereo vision for 3-D tracking and localization and monocular vision for detection. The authors of [161] proposed a scheme for vehicle detection using a classifier on the monocular plane, an estimation of the ground surface using disparity map, and tracking in the stereo-vision domain with the help of extended Kalman Filter (KF). In a related work [162], initial training of AdaBoost detectors for multiple vehicle views was carried out, and then verification of candidate regions was observed by finding the peaks in disparity range.

### 5.2. Active Sensors

The active sensors first radiate signals and then sense the reflected signals to identify other vehicles and obstacles. Their examples include radar-based [163] and Laser-based [164] sensors. To detect and track the obstacles in front of a vehicle, the authors of [165] used Pulse Doppler radar framework with sensors installed in the front-lower part of a vehicle. The distance between the target and the vehicle was calculated by examining echoes of radar signals. The developed system’s performance was also observed at various distance ranges and under different weather conditions. For detection of an approaching vehicle, a detection scheme based on radar was proposed in [166] which exploited the sparsity of the cyclic autocorrelation. This scheme showed good simulation results, but its practical demonstration was not provided. Another study [167] developed a radar-based driver safety system for an actual multi-lane system using discrete time signal processing. With 200 classification tests, a high accuracy of 90% was achieved for vehicle speed detection. Moreover, the results for the developed system under different situations such as low-light conditions which correspond to fog and rain were satisfactory.

The detection and tracking systems based on Lidar and laser transmit and receive UV, visible, and IR waves of the EM spectrum. The returning wave collected at the receiver provides information about the distance of the object. Commonly available 1-D and 2-D Lidar sensors are more economical than radars. Laser scanners, which are an extended version of laser range finders, adopt the time-of-flight principle to compute the distance to an object. A scheme for detection and classification of vehicles is developed in [164] by using a vehicle-mounted Laser scanner. The developed system was tested under multiple driving conditions (e.g., city and highways traffic) with three different Laser scanners, and resulted in good accuracy. Advanced laser scanners acquire data with high a scanning rate at a high spatial resolution. Table 3 provides a summary of the commonly available active and passive sensors.

### 5.3. Combination or Fusion of Sensors

The modern trend is shifting towards the application of multiple sensors combined to produce a comprehensive set of reliable and secure results [178,186,187]. When used as a combination or fusion, either one sensor detects and the other validates, or both sensors perform detection at the same time and then validate their results [177,185].

#### 5.3.1. Vision and Radar

Recently, the fusion of vision and radar sensors for vehicle detection and tracking received increased attention. In this combination, radar is primarily used for evaluating the distance or regions of interest whereas recognition is performed by pattern recognition algorithms applied to visual data. As observed in [184], railings’ location was estimated by radar data, and vehicles were detected by the vertical symmetry attribute of certain region in the images [184]. In other studies [155,159], optical features of images, such as edge and symmetry, were used to detect vehicles, and KF was implemented on radar data for ranging and tracking of the vehicles. In [171], the authors used classifiers, such as Gabor, a histogram of oriented gradients, and Haar on images data to detect the vehicles, and calculated the range using radar data. The study carried out in [179] analyzed the input image for salient locations using multiple features such as intensity, orientation, and color. Once the vehicle is detected, its distance is computed using radar and vision fused data. Similar techniques were adopted in other studies, such as [183].

#### 5.3.2. Vision and Lidar

Typically, in these experiments, detection and tracking are initially performed using Lidar data, and then Lidar and camera are simultaneously utilized for classification. Fusion of monocular vision with Lidar is reported in [178]. In [177], potential obstacles were initially discovered by multi-layer Lidar, and then a stereo vision system was employed to confirm their existence. The authors of [176] utilized a multi-sensor scheme using camera, radar, and lidar technologies to acquire widely overlapping fields of view. The complete assembly consisted of two separate laser scanners, multiple short-range radars mounted on the vehicles’ sides, and three long-range sensors (i.e., radar, stereo-vision, and laser) covering the vehicle’s front. Consequently, the obstacle map developed through fusion of multiple sensors data produced precise and more reliable results than any of individual sensors’ results. Such an assembly, however, requires a healthy budget.

#### 5.3.3. Vision and Sound

In this combination, direction-of-arrival of other vehicles is estimated through acoustic data processing whereas target location is calculated using cameras [175].

#### 5.3.4. Radar and Lidar

Though involving higher cost, this combination produces improved detection and tracking results. For example, in [174], a system based on combined information collected through radar and lidar is proposed. The state estimation was carried out using Bayesian methods, and tracking data produced by two independent systems was combined to produce improved results.

#### 5.3.5. Other Combinations

Studies have also proposed other combinations of active and passive sensors. For example, a combination of sound sensor, radar, stereo and IR camera is proposed in [173] for detection and monitoring of motorcycles.

## 6. DMAS in Modern Vehicles

As explained in Section 2, the driver’s role in the driving process is typically divided into three activities, viz. situation awareness, decision, and action. High-end models of modern vehicles are equipped with DMAS which assist the driver in these activities, as shown in Table 4. A brief survey of literature and systems dedicated to assisting the drivers in safe and comfortable driving is presented below.

### 6.1. Assistance in Situation Awareness

Improving a driver’s awareness about the situation is among the basic themes of DMAS. The necessary information about the driving environment is provided to the driver to make a well-judged and timely decision [193,194,195,196,197,198,199,200,201]. In this regard, vision enhancement is a major subject as most driving-related information is collected through the eyes [202]. Considering the vision systems which serve as an extension to the human eye, there are two main categories of vision enhancement techniques:

1. Inside-vehicle screens: A typical example of such systems is the rear-view camera extensively used for parking. Other examples are infrared cameras which dynamically capture the scenes ahead of the vehicle, and relay them to the driver in an enhanced form. Display of such infrared cameras is usually located on top of the dashboard in front of the driver. The inside-vehicle screens deliver additional information to the drivers that is usually invisible, and sometimes irrelevant as well. Consequently, it increases recognition burden for the drivers. These displays always divert drivers’ attention regardless of their position in the vehicle.
2. Outside-vehicle lighting arrangement: These systems dynamically tweak the intensity and range of vehicle lights to attain a continuous transition between high/low beam illuminations or differentiate possible obstructions for the drivers. Marking Light [196] from Volkswagen is an example of such systems. Comparative to inside-vehicle screens, the outside-vehicle lighting systems are considered to be more natural and easier for the drivers, but not free of intrusions [203,204].

Information provided by these systems to the drivers is typically not detected by human eyes (e.g., possible pedestrians [201] or additional visual information collected from nearby vehicles [194,199]). However, inputting this information contains the risk of confusing the driver’s recognition. Occasionally, this could be very dangerous as the resultant confusion can disorganize the driver’s recognition system all of a sudden [168,194].

Enhancing the drivers’ awareness in a time of disturbances caused by the weather and environment is another interesting topic of further research. For instance, detection of raindrops and rain speed is used in speed adjustment of smart-wipers systems to provide a clear view for drivers during rain [193,197,198]. Similarly, resolving how to handle the irritating and often dangerous high beam light glare from the passing vehicles is still an unsolved problem. Introduction of intelligent headlight adjustment systems [200] seems to be a standard solution. Such a system decreases the intensity of headlights when other approaching vehicles are identified. However, vehicles without intelligent headlight adjustment systems still pose the danger to the drivers driving a vehicle with such a system. The authors’ point of view in this regard is to standardize such a system in future vehicles.

### 6.2. Assistance in Decision Making

In comparison to situation awareness enhancement, assistance in decision making is the next level of DMAS as it provides guidance to drivers in a loud and clear manner [205,206,207,208,209,210,211,212,213,214,215,216,217,218,219,220]. There is an increased interest in how a vehicle communicates with its drivers and vice-versa [205,206,207,208]. From the literature survey, the following main categories of decision enhancement systems are identified:

1. Audio system (e.g., voice navigation and warning)
2. Video or visual system (e.g., displays)
3. Miscellaneous (a combination of above two, vibration, etc.)

The audio systems deliver lesser information than video systems in several conditions and are comparatively less distracting. In general, only rout suggestions such as left or right turns are efficiently conveyed by audio guidance systems. Additionally, audio warning systems are also useful for drowsy or drunk drink drivers to convey emergency action orders because of their inattentiveness to visual warnings.

The visual displays offer more useful information with productive features but also distract the driver’s attention [209]. An example of such systems is a 3-D navigation system getting increased popularity. To the authors of this survey, additional experimentation and data are needed to assess the optimal tradeoff for users of such systems as they augment the drivers’ recognition burden.

Another issue arose due to limited space of the dashboard is how to place, adjust, and organize the vehicle’s classic meters (e.g., temperature indicator, speedometer) and modern visual displays. As proposed in [212,213], a multi-function display that represents maximum information by merging several separate displays and controls into a single graphical user interface will get popularity in future vehicles. However, shifting to multi-function displays from the conventional dashboards will certainly involve additional usage concerns and more challenges in human driver ergonomics [205,206,207,208,217]. Similarly, due to the difference in driving conditions (e.g., between highway and city driving [210]), development of an adaptive interface to accommodate such diversities is still an open challenge.

### 6.3. Assistance in Action Performing

Recent developments in the field of human-machine interaction and cooperation enable the modern DMAS to move one step further by assisting the driver in the action performing stage [221,222,223,224,225,226,227,228,229,230,231,232]. An example of action-assistance is the reverse steering feature offered by Ford as shown in Table 4. As explained in [221,233], action-assistance has several levels of complexity and driver-vehicle interaction. The lateral steer-by-wire control system, available in many vehicles, represents a simple level [222,223,224,225,226,234]. These systems pacify the steering control by filtering out the inaccuracies and disturbances associated with human driving behaviors. Similarly, the longitudinal brake-by-wire control analyzes the pattern of an individual driver’s car-following and pedal usage style to offer a tailored driving experience [227,228,229,230,235]. However, appropriate and timely adjustment of DMAS is a challenge as human drivers slowly and constantly change their driving styles [228]. The higher level action-assistance type controls include advanced lane departure assistance system [231] and higher-level brake control system [236].

The time and to what extent DMAS can take control is a hot topic in this research [236,237,238,239,240,241,242,243]. Though the automation of driving process has an attractive side, yet, over trusting it may result in surprises.

### 6.4. Future Trends

DMAS have promising safety-enhancing features that simplify the driving process, reduce sources of driver distraction, and inattention that often lead to accidents. With DMAS support, it is expected that drivers and passengers will find safer roadways, countering deadly trends in road accidents. State-of-the-art DMAS shall combine the features and capabilities built on advanced and diverse technologies. To the best of the authors’ understanding, the future of DMAS lies in the following key trends [244,245,246,247,248]:

- **Connectivity:** Communication networks are becoming an integral part of both external and in-vehicle connectivity as vehicle-related digital-data grows substantially. In addition to their assistance in crucial systems such as braking systems and tire-pressure monitoring, wireless networks provide superior flexibility for regular automotive communications protocols. The development of highly integrated wireless devices offers a flexible foundation for services that keep drivers informed about vehicle status and road conditions. Moreover, new technologies like the Internet of Things can connect smart devices with vehicles’ communication system to deliver more sophisticated services.
- **Sensors:** DMAS necessitate a wide-ranging set of sensors for monitoring the vehicles’ surroundings and drivers’ condition. The modern trend is toward signal-chain integration and enhanced sensor fusion, which combine the output of various sensors to provide more extrapolative information. For example, by merging sensors’ data from tire-pressure sensors, anti-lock braking system, acceleration sensors, and electronic-stability control, the researchers are developing systems that can predict a loss of friction between the tire and the road.
- **Embedded vision:** Vision systems are critical to identify and track the possible hazards. These systems provide critical input for high-level warning functions, including unobserved vehicles or lane drift and support other services such as automatic parking and traffic sign recognition.
- **Automotive systems infrastructure:** The modern vehicles’ control is significantly dependent on the increased integration of smart sensors. This situation requires an improved system foundation in DMAS architectures as well as throughout the vehicle system design. With several processors scattered throughout the vehicle, the necessity for a stable design platform is evident, as indicated in ISO standards [249]. There is a growing list of real-time operating systems, middleware, and development tools designed to support the ISO 26262 international functional safety standard for road vehicles.
- **Human-machine interface (HMI) design:** The success of DMAS eventually lies in distraction-free interaction for the driver, though improved vision, sensors, and connectivity. For an improved driving experience itself, the most promising trend is perhaps the application of advanced HMI technologies. The touchscreen technology may assist drivers when the vehicle is parked or help passengers. Touch-free HMI systems offer the mechanisms for driver interaction without requiring hands off the steering wheel.

## 7. Discussion and Conclusions

**Figure 6:**
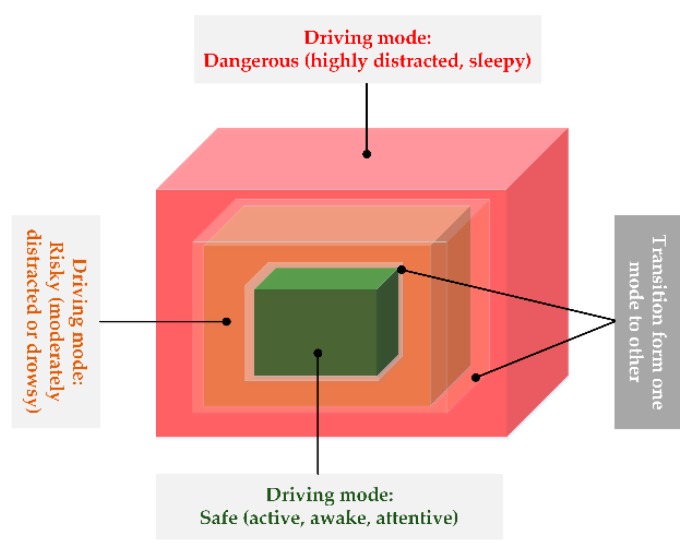
Typical driving modes of a driver and transition stages.

The benefits of road transportation for individual and society are accompanied by certain losses in the form of life, property, and environmental pollution. For decades, there have been several plans, including DMAS, to improve the driving process and reduce the losses. Based on the literature review, this work classified three main causes of driving accidents, viz. distraction, fatigue, and aggressive driving behavior. In this survey, the authors reviewed DMAS in a comprehensive way by considering factors associated with the driver, vehicle, and driving environment. The driver’s attentiveness is the primary element for safe driving. Distraction and fatigue are the main causes of road accidents. The studies reveal that several biological and physiological measurements can accurately detect a driver’s mental engagement. Similarly, the application of modern classification techniques on the data obtained from the vehicle’s instrumentation provides a good measure of driving behavior. The detection of nearby vehicles is also an important feature of DMAS to avoid any possible collisions. The study areas involved in DMAS are closely interlinked, and it is often hard to draw a clear boundary between two areas. Similarly, as shown in Figure 6, the models developed for DMAS usually consider a transition band for drivers when moving from one state to another (e.g., from alert to drowsy). The modern classification techniques based on machine learning algorithms prove useful in handling such situations.

In addition to alerting a driver on the verge of a mistake, the modern DMAS also offer assistance at decision making and action taking stages. However, such systems did not receive mass adoption and are still under research. Before the spread of such systems, however, not only an appropriate regulatory charter must be defined, but meaningful research is also required. The associated main challenges are quick and correct decisions by the machines which are typically based on the machines’ programming. The transferring of human drivers’ experience to machine cognition is an important step in the field.

The acceptance of these intelligent and vulnerable systems by its end users is not a simple task. This definitely requires functional reliability, safety, and transparency with respect to autonomous performance. At the same time, the design of user-friendly ergonomic human-machine interface must not be ignored. The authors propose that these aspects call for a considerable amount of in-depth multidisciplinary research for several years.

## Figures and Tables

**Table 1:** A summary of studies related to the driver’s attention monitoring based on biological and physiological parameters.

Study Area	Signal	Typical Range	Correlation with Fatigue		Detection Accuracy	References	Commercially Available Sensors
			Positive	Negative			
Fatigue detection	ECG	50 µV–50 mV [71] 0.05 Hz–100 Hz		Heart rate	96% (30 volunteers)	[71,72,75,101,104]	Omron, Flex Sensors, EPI mini, Alivecor System and ECG Check, Ambulatory ECG, Drypad Sensors, NeuroSky’s Dry Sensor, Quasar sensors
			HRV				
			HF	VLF, LF, LF/HF			
				RR			
Fatigue and distraction detection	EEG	2 µV–10 µV [71] 10 Hz–2 kHz	α, θ Bands Powers	β Band Power	96.7% [105] (6 volunteers)	[106,107,108,109,110]	Drypad Sensors, Imotive Headset, MindWave Headsets, NeuroSky’s Dry Sensor, Quasar Sensors, Flex Sensors
			P300 Latency	P300 Amplitude			
				Entropy			
Detection of alertness	EOG	0.05 mV–3.5 mV [61] 0.1 Hz–100 Hz [71]	Blink Duration		81.7% (20 volunteers)	[89,111,112,113,114,115]	SMI Eye Tracking Glasses, NeuroSky’s Dry Sensor, Google glass, Comnoscreen, ASL Eye Tracking Glasses
			Blink Frequency Time				
			Lid Reopening				
				Blink Amplitude			
			PERCLOS				
				Eye Movements			
Fatigue detection	EMG	20 µV–10 mV [71] 10 Hz–10 kHz		EMG Amplitude	94% [67] (4 volunteers)	[67,91,96,97,98,115,116]	SX230, Neuronode, NeuroSky’s Dry Sensor, Trigno Mini Sensor, Quasar Sensors
			Centre frequency shift towards lower frequency region				
Fatigue detection	EDA	10 kΩ–10 MΩ 1.76 V–0.14 V	Skin Resistance	EDA	80% [101] (13 volunteers)	[117]	Shimmer 3, Empatica wristband, Grove — GSR
Fatigue detection	ST	89.6°F–95°F [118]		ST		[118]	YSI 400 Series Temperature Probe, MAXIM30205

**Table 2:** A survey of work related to driving style recognition.

Levels	Description of Levels	Objective	Inputs	Reference
2	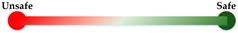	Safety	Speed, fuel consumption, accelerometer, throttle	[123,126,127,128,129,130,131,132]
3	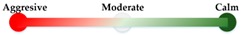	Safety	Brake, throttle, car following	[129,133,134,135]
4	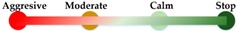	Safety	Jerk	[136]
4	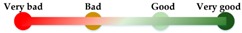	Behavioral analysis	Sharp turn, acceleration, deceleration	[137,138]
5–7	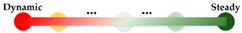	Behavioral analysis	Acceleration, speed	[139]
4	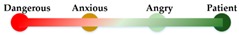	Behavioral analysis	Personality features	[140]
(−1,1)	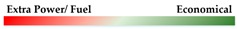	Fuel economy	Kinetic energy, accelartion, speed	[141,142,143]
(−1,1)	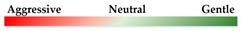	Behavioral analysis, safety	Brake, speed, turn	[144]

**Table 3:** A survey of sensors applicable in the field of vehicle detection and tracking systems.

Type	Typical Range	Description		References	Specific Sensor
		Advantages	Disadvantages		
Acoustic	Variable	An economical solution, Real time Omni—directional microphone,	Noise sensitive, Short range, Interference problem	[153,168]	SONY ECM-77B
Radar	175 m	Robust in foggy or rainy day, and during night time, Measure distance directly with less computing resources, Longer detection range than acoustic, and optical sensor	Classification issue, More Power consumption than acoustic and optical sensor, Interference problem, Higher cost than Acoustic sensors	[163,165,166]	Delphi Adaptive Cruise Control
Laser/Lidar	120 m	Independent of weather conditions, Longer detection range than acoustic and optical sensor, Modern lidar/laser scanners acquire high resolution and 3D information	More Power consumption than other sensors, High speed 3D scanners are expensive Road infrastructure dependency	[164,169,170]	Velodyne HDL-64E Laser Rangefinder (31D LIDAR)
	80 m				SICK LMS5l-l0l00 (2D)
Optical (camera)	100 m (day) l2 m (night)	Accumulate data in nonintrusive way, Higher resolution and wider view angle, Low cost, easier to install and maintain, Extensive information in images, Independent of any modifications to the road infrastructure	Requires more computing resources to process the images, Image quality depends on lighting and weather conditions	[154,155,157,158,171,172]	SV-625B
Fusion	Variable	Maximum information of surroundings, Increased system robustness and reliability, Broadens the sensing capabilities,	Expensive, Separate algorithms for each Sensor	[153,169,171,173,174,175,176,177,178,179,180,181,182,183,184,185]	Not Applicable

**Table 4:** A summary of DMAS available in modern vehicles.

Company	Technology	Category	Monitoring System/Detection Parameters/Warning System	Important Features	Reference
Audi	Audi pre sense (driver assistance system)	Car-based	Far infrared system, Camera, Radar, Thermal camera/Lane position, Proximity detection/Audio, display, vibration	Collision avoidance assist Sunroof and windows closing High beam assist Turn assist Rear cross-path assist Exit assist (to warn door opening when a nearby car passes) Traffic jam assist Night vision	[188]
BMW	BMW Drive Assist (driver assistance system)	Car-based	Radar, Camera, Thermal camera/Lane position, Proximity detection/Audio, display, vibration	Lane change warning Night vision Steering and lane control system for semi-automated driving Crossroad warning Assistive parking	[189]
Toyota	Toyota Safety Sense (Driver moniting system)	Driver-based	Radar, Charge-coupled camera/Eye tracking and head motion/Audio, display	Advanced obstacle detection system Pre-Collision System Lane Departure Alert Automatic High Beams Dynamic Radar Cruise Control Pedestrian Detection	[190]
Mercedez-Benz	Mercedez-Benz Pre-safe Technology (Attention assist)	Car-based	Radar, Camera, Sensors on the steering column/Steering wheel movement and speed/Audio, display	Driver’s profile and behaviour Accident Investigation Pre-Safe Brake and Distronic Plus Technology Night View Assist Plus Active Lane Keeping Assist and Active Blind Spot Monitoring Adaptive High Beam Assist Attention assist	[191]
Ford	Ford Safe and Smart (Driver alert control)	Car based	Radar, Camera, Steering sensors/Lane position, Proximity detection/Audio, display, vibration	Lane-Keeping System Adaptive cruise control Forward collision warning with brake support Front rain-sensing windshield wipers. Auto high-beam headlamps Blind Spot Information System Reverse steering	[192]

## References

[1] WHO|Decade of Action for Road Safety 2011–2020. WHO 2018. https://www.who.int/roadsafety/decade_of_action/en/.

[2] Traffic S. (2006). Vision Zero on the Move. https://ec.europa.eu/transport/road_safety/sites/roadsafety/files/pdf/20151210_1_sweden.pdf.

[3] Ranney T.A., Garrott W.R., Goodman M.J. (2001). NHTSA Driver Distraction Research: Past, Present, and Future.

[4] Young M.A.R., John D.L., Kristie Y., John D.L., Kristie (2019). Driver Distraction: Theory, Effects, and Mitigation.

[5] WHO|Global Status Report on Road Safety 2013. WHO 2015. https://www.who.int/violence_injury_prevention/road_safety_status/2013/en/.

[6] WHO|World Report on Road Traffic injury Prevention. WHO 2014. https://www.who.int/violence_injury_prevention/publications/road_traffic/world_report/en/.

[7] Organization W.H. (2009). Global Status Report on Road Safety: Time for action. http://www.who.int/iris/handle/10665/44122.

[8] Di Flumeri G., Borghini G., Aricò P., Sciaraffa N., Lanzi P., Pozzi S., Vignali V., Lantieri C., Bichicchi A., Simone A., Ayaz H., Dehais F. (2019). Chapter 20—EEG-Based Mental Workload Assessment During Real Driving: A Taxonomic Tool for Neuroergonomics in Highly Automated Environments. Neuroergonomics.

[9] Faure V., Lobjois R., Benguigui N. (2016). The effects of driving environment complexity and dual tasking on drivers’ mental workload and eye blink behavior. Transp. Res. Part F Traffic Psychol. Behav..

[10] Sakakibara K. Evaluation of driver’s state of tension. Proceedings of the HCII 11th International Conference on Human-Computer Interaction.

[11] Bitkina O.V., Kim J., Park J., Park J., Kim H.K. (2019). Identifying Traffic Context Using Driving Stress: A Longitudinal Preliminary Case Study. Sensors.

[12] Li L., Wen D., Zheng N., Shen L. (2012). Cognitive Cars: A New Frontier for ADAS Research. IEEE Trans. Intell. Transp. Syst..

[13] Mukhtar A., Xia L., Tang T.B. (2015). Vehicle detection techniques for collision avoidance systems: A review. IEEE Trans. Intell. Transp. Syst..

[14] Endsley M.R. (1995). Toward a Theory of Situation Awareness in Dynamic Systems. Hum. Factor.

[15] Stutts J.C., Reinfurt D.W., Staplin L., Rodgman E. (2001). The Role of Driver Distraction in Traffic Crashes.

[16] Ranney T., Mazzae E., Garrott R., Goodman M., Administration N.H.T.S. Driver distraction research: Past, present and future. Proceedings of the 17th International Technical Conference of Enhanced Safety of Vehicles.

[17] Zhao Y., Görne L., Yuen I.-M., Cao D., Sullman M., Auger D., Lv C., Wang H., Matthias R., Skrypchuk L. (2017). An Orientation Sensor-Based Head Tracking System for Driver Behaviour Monitoring. Sensors.

[18] Khandakar A., Chowdhury M.E.H., Ahmed R., Dhib A., Mohammed M., Al-Emadi N.A.M.A., Michelson D. (2019). Portable System for Monitoring and Controlling Driver Behavior and the Use of a Mobile Phone While Driving. Sensors.

[19] Fitch G.M., Soccolich S.A., Guo F., McClafferty J., Fang Y., Olson R.L., Perez M.A., Hanowski R.J., Hankey J.M., Dingus T.A. (2013). The Impact of Hand-Held and Hands-Free Cell Phone Use on Driving Performance and Safety-Critical Event Risk.

[20] Husen M.N., Lee S., Khan M.Q. Syntactic pattern recognition of car driving behavior detection. Proceedings of the 11th International Conference on Ubiquitous Information Management and Communication.

[21] Liao Y., Li S.E., Wang W., Wang Y., Li G., Cheng B. (2016). Detection of driver cognitive distraction: A comparison study of stop-controlled intersection and speed-limited highway. IEEE Trans. Intell. Transp. Syst..

[22] Hogsett J., Kiger S. (2006). Driver Workload Metrics Project: Task 2 Final Report.

[23] Liao Y., Li G., Li S.E., Cheng B., Green P. (2018). Understanding Driver Response Patterns to Mental Workload Increase in Typical Driving Scenarios. IEEE Access.

[24] Harbluk J.L., Noy Y.I., Trbovich P.L., Eizenman M. (2007). An on-road assessment of cognitive distraction: Impacts on drivers’ visual behavior and braking performance. Accid. Anal. Prev..

[25] Rantanen E.M., Goldberg J.H. (1999). The effect of mental workload on the visual field size and shape. Ergonomics.

[26] Harbluk J.L., Noy Y.I., Eizenman M. (2002). The Impact of Cognitive Distraction on Driver Visual Behaviour and Vehicle Control.

[27] Hayhoe M.M. (2004). Advances in relating eye movements and cognition. Infancy.

[28] May J.G., Kennedy R.S., Williams M.C., Dunlap W.P., Brannan J.R. (1990). Eye movement indices of mental workload. Acta Psychol..

[29] Miyaji M., Kawanaka H., Oguri K. Driver’s cognitive distraction detection using physiological features by the adaboost. Proceedings of the 12th International IEEE Conference on Intelligent Transportation Systems.

[30] Liang Y., Lee J.D. (2010). Combining cognitive and visual distraction: Less than the sum of its parts. Accid. Anal. Prev..

[31] Zhang H., Smith M., Dufour R. (2008). A Final Report of Safety Vehicles Using Adaptive Interface Technology (Technical Report Phase II: Task 7C): Visual Distraction.

[32] Itoh M. Individual differences in effects of secondary cognitive activity during driving on temperature at the nose tip. Proceedings of the International Conference on Mechatronics and Automation.

[33] Wesley A., Shastri D., Pavlidis I. A novel method to monitor driver’s distractions. Proceedings of the CHI’10 Extended Abstracts on Human Factors in Computing Systems.

[34] Berka C., Levendowski D.J., Lumicao M.N., Yau A., Davis G., Zivkovic V.T., Olmstead R.E., Tremoulet P.D., Craven P.L. (2007). EEG correlates of task engagement and mental workload in vigilance, learning, and memory tasks. Aviat. Space Environ. Med..

[35] Ranney T.A. (2008). Driver Distraction: A Review of the Current State-of-Knowledge.

[36] Zhou H., Itoh M., Inagaki T. Influence of cognitively distracting activity on driver’s eye movement during preparation of changing lanes. Proceedings of the SICE Annual Conference.

[37] Carsten O., Brookhuis K. (2005). Issues arising from the HASTE experiments. Transp. Res. Part F Traffic Psychol. Behav..

[38] Council E.T.S. (2001). The Role of Driver Fatigue in Commercial Road Transport Crashes.

[39] Brill J.C., Hancock P.A., Gilson R.D. Driver Fatigue: Is Something Missing?. Proceedings of the Driving Assessment Conference.

[40] (2001). Driver Fatigue and Road Accidents: A Literature Review and Position Paper.

[41] Borrelli I. (2007). Safety of professional drivers: Literature review about prevention measures linked to sleeping. Giornale Italiano di Medicina del Lavoro ed Ergonomia.

[42] Mast T.M., Jones H.V., Heimstra N.W. (1966). Effects of Fatigue on Performance in a Driving Device.

[43] Kahneman D. (1973). Attention and Effort.

[44] Yabuta K., Iizuka H., Yanagishima T., Kataoka Y., Seno T. (1985). The Development of Drowsiness Warning Devices.

[45] Elling M., Sherman P. Evaluation of steering wheel measures for drowsy drivers. Proceedings of the 27th International Symposium on Automotive Technology and Automation.

[46] Dingus T., Hardee L., Wierwille W. (1985). Development of Impaired Driver Detection Measures.

[47] Zhong Y.-J., Du L.-P., Zhang K., Sun X.-H. Localized energy study for analyzing driver fatigue state based on wavelet analysis. Proceedings of the International Conference on Wavelet Analysis and Pattern Recognition.

[48] Skipper J.H. (1985). An Investigation of Low-Level Stimulus-Induced Measures of Driver Drowsiness.

[49] Stein A.C. (1995). 15 Detecting fatigued drivers with vehicle simulators. Fatigue and Driving: Driver Impairment, Driver Fatigue, and Driving Simulation.

[50] Riemersma J.B.J., Sanders A.F., Wildervanck C., Gaillard A.W., Mackie R.R. (1977). Performance Decrement during Prolonged Night Driving. Vigilance.

[51] Safford R.R., Rockwell T.H. (1967). Performance decrement in twenty-four hour driving. Highway Research Record.

[52] (1998). PERCLOS: A Valid Psychophysiological Measure of Alertness as Assessed by Psychomotor Vigilance.

[53] Li G., Chung W.-Y. (2014). Estimation of Eye Closure Degree Using EEG Sensors and Its Application in Driver Drowsiness Detection. Sensors.

[54] Daza I.G., Bergasa L.M., Bronte S., Yebes J.J., Almazán J., Arroyo R. (2014). Fusion of Optimized Indicators from Advanced Driver Assistance Systems (ADAS) for Driver Drowsiness Detection. Sensors.

[55] Eskandarian A., Sayed R., Delaigue P., Mortazavi A., Blum J. (2007). Advanced Driver Fatigue Research.

[56] Li Z., Chen L., Peng J., Wu Y. (2017). Automatic Detection of Driver Fatigue Using Driving Operation Information for Transportation Safety. Sensors.

[57] Mandal B., Li L., Wang G.S., Lin J. (2017). Towards detection of bus driver fatigue based on robust visual analysis of eye state. IEEE Trans. Intell. Transp. Syst..

[58] Nilsson T., Nelson T.M., Carlson D. (1997). Development of fatigue symptoms during simulated driving. Accid. Anal. Prev..

[59] Mohan D. Analysis of road traffic fatality data for Asia. Proceedings of the 9th International Conference of Eastern Asia Society for Transportation Studies.

[60] Kataoka H., Satoh Y., Aoki Y., Oikawa S., Matsui Y. (2018). Temporal and Fine-Grained Pedestrian Action Recognition on Driving Recorder Database. Sensors.

[61] Thorslund B. (2004). Electrooculogram Analysis and Development of a System for Defining Stages of Drowsiness.

[62] Yeo M.V.M., Li X., Shen K., Wilder-Smith E.P.V. (2009). Can SVM be used for automatic EEG detection of drowsiness during car driving?. Saf. Sci..

[63] Shen K.-Q., Li X.-P., Ong C.-J., Shao S.-Y., Wilder-Smith E.P.V. (2008). EEG-based mental fatigue measurement using multi-class support vector machines with confidence estimate. Clin. Neurophysiol..

[64] Chin-Teng L., Ruei-Cheng W., Sheng-Fu L., Wen-Hung C., Yu-Jie C., Tzyy-Ping J. (2005). EEG-based drowsiness estimation for safety driving using independent component analysis. IEEE Trans. Circuits Syst. I Regul. Pap..

[65] Skinner B.T., Nguyen H.T., Liu D.K. Classification of EEG Signals Using a Genetic-Based Machine Learning Classifier. Proceedings of the 2007 29th Annual International Conference of the IEEE Engineering in Medicine and Biology Society.

[66] Liu J., Zhang C., Zheng C. (2010). EEG-based estimation of mental fatigue by using KPCA–HMM and complexity parameters. Biomed. Signal Process. Control.

[67] Hwang T., Kim M., Hong S., Park K.S. Driver drowsiness detection using the in-ear EEG. Proceedings of the 2016 38th Annual International Conference of the IEEE Engineering in Medicine and Biology Society (EMBC).

[68] Rahim H.A., Dalimi A., Jaafar H. (2015). Detecting drowsy driver using pulse sensor. J. Teknol..

[69] Furman G.D., Baharav A., Cahan C., Akselrod S. Early detection of falling asleep at the wheel: A Heart Rate Variability approach. Proceedings of the 2008 Computers in Cardiology.

[70] Hartley L.R., Arnold P.K., Smythe G., Hansen J. (1994). Indicators of fatigue in truck drivers. Appl. Ergon..

[71] Wilson G.F., O’Donnell R.D., Hancock P.A., Meshkati N. (1988). Measurement of Operator Workload with the Neuropsychological Workload Test Battery. Advances in Psychology.

[72] Mulder G., Mulder—Hajonides Van Der Meulen W.R.E.H. (1973). Mental Load and the Measurement of Heart Rate Variability. Ergonomics.

[73] Kalsbeek J.W.H. (1968). Measurement of mental work load and of acceptable load: possible applications in industry. Int. J. Prod. Res..

[74] Vicente J., Laguna P., Bartra A., Bailón R. (2016). Drowsiness detection using heart rate variability. Med. Biol. Eng. Comput..

[75] Lee D.H., Park K.S. (1990). Multivariate analysis of mental and physical load components in sinus arrhythmia scores. Ergonomics.

[76] Shinar Z., Akselrod S., Dagan Y., Baharav A. (2006). Autonomic changes during wake–sleep transition: A heart rate variability based approach. Auton. Neurosci..

[77] Toledo E., Gurevitz O., Hod H., Eldar M., Akselrod S. (2003). Wavelet analysis of instantaneous heart rate: A study of autonomic control during thrombolysis. Am. J. Physiol. Regul. Integr. Comp. Physiol..

[78] Sun Y., Yu X., Berilla J., Liu Z., Wu G. An in-vehicle physiological signal monitoring system for driver fatigue detection. Proceedings of the 3rd International Conference on Road Safety and Simulation Purdue University Transportation Research Board.

[79] Yue C. (2011). EOG Signals in Drowsiness Research. Master’s Thesis.

[80] Andreassi J.L. (2010). Psychophysiology: Human Behavior and Physiological Response.

[81] Schleicher R., Galley N., Briest S., Galley L. (2008). Blinks and saccades as indicators of fatigue in sleepiness warnings: Looking tired?. Ergonomics.

[82] Thum Chia C., Mustafa M.M., Hussain A., Hendi S.F., Majlis B.Y. Development of vehicle driver drowsiness detection system using electrooculogram (EOG). Proceedings of the 1st International Conference on Computers, Communications, & Signal Processing with Special Track on Biomedical Engineering.

[83] Sirevaag E.J., Stern J.A., Boucsein W. (2000). Ocular measures of fatigue and cognitive factors. Engineering psychophysiology: Issues and applications.

[84] Svensson U. (2004). Blink Behaviour Based Drowsiness Detection: Method Development and Validation.

[85] Ebrahim P. (2016). Driver Drowsiness Monitoring Using Eye Movement Features Derived from Electrooculography.

[86] Shin D.U.K., Sakai H., Uchiyama Y. (2011). Slow eye movement detection can prevent sleep-related accidents effectively in a simulated driving task. J. Sleep Res..

[87] Bosch T., de Looze M.P., Kingma I., Visser B., van Dieën J.H. (2009). Electromyographical manifestations of muscle fatigue during different levels of simulated light manual assembly work. J. Electromyogr. Kinesiol..

[88] Reaz M.B.I., Hussain M.S., Mohd-Yasin F. (2006). Techniques of EMG signal analysis: Detection, processing, classification and applications. Biol. Proc. Online.

[89] Karlsson S., Jun Y., Akay M. (2000). Time-frequency analysis of myoelectric signals during dynamic contractions: A comparative study. IEEE Trans. Biomed. Eng..

[90] Fu R., Wang H. (2014). Detection of driving fatigue by using noncontact EMG and ECG signals measurement system. Int. J. Neural Syst..

[91] Chen R. (2013). Sitting Behaviour-Based Pattern Recognition for Predicting Driver Fatigue. Ph.D. Thesis.

[92] Balasubramanian V., Adalarasu K. (2007). EMG-based analysis of change in muscle activity during simulated driving. J. Bodyw. Mov. Ther..

[93] Khushaba R.N., Kodagoda S., Lal S., Dissanayake G. (2011). Driver Drowsiness Classification Using Fuzzy Wavelet-Packet-Based Feature-Extraction Algorithm. IEEE Trans. Biomed. Eng..

[94] Tan Z.X., Foong R., Ang K.K. Determining mechanical and electromyographical reaction time in a BCI driving fatigue experiment. Proceedings of the 10th International Conference on Information, Communications and Signal Processing (ICICS).

[95] Lin M.-I., Liang H.-W., Lin K.-H., Hwang Y.-H. (2004). Electromyographical assessment on muscular fatigue—an elaboration upon repetitive typing activity. J. Electromyogr. Kinesiol..

[96] Luttmann A., JĀGer M., SÖKeland J., Laurig W. (1996). Electromyographical study on surgeons in urology. II. Determination of muscular fatigue. Ergonomics.

[97] Kumar D.K., Pah N.D., Bradley A. (2003). Wavelet analysis of surface electromyography. IEEE Trans. Neural Syst. Rehabil. Eng..

[98] Villarejo M.V., Zapirain B.G., Zorrilla A.M. (2012). A Stress Sensor Based on Galvanic Skin Response (GSR) Controlled by ZigBee. Sensors.

[99] Haag A., Goronzy S., Schaich P., Williams J. (2004). Emotion Recognition Using Bio-Sensors: First Steps towards an Automatic System.

[100] Bando S., Oiwa K., Nozawa A. (2017). Evaluation of dynamics of forehead skin temperature under induced drowsiness. IEEJ Trans. Electr. Electron. Eng..

[101] Awais M., Badruddin N., Drieberg M. (2017). A Hybrid Approach to Detect Driver Drowsiness Utilizing Physiological Signals to Improve System Performance and Wearability. Sensors.

[102] Yu S., Li P., Lin H., Rohani E., Choi G., Shao B., Wang Q. Support Vector Machine Based Detection of Drowsiness Using Minimum EEG Features. Proceedings of the 2013 International Conference on Social Computing.

[103] Xue-Qin H., Zheng W., Lu B. Driving fatigue detection with fusion of EEG and forehead EOG. Proceedings of the International Joint Conference on Neural Networks (IJCNN).

[104] Keselbrener L., Akselrod S. (1996). Selective discrete Fourier transform algorithm for time-frequency analysis: Method and application on simulated and cardiovascular signals. IEEE Trans. Biomed. Eng..

[105] Kurt M.B., Sezgin N., Akin M., Kirbas G., Bayram M. (2009). The ANN-based computing of drowsy level. Expert Syst. Appl..

[106] Li G., Chung W.-Y. (2015). A Context-Aware EEG Headset System for Early Detection of Driver Drowsiness. Sensors.

[107] Lawhern V., Kerick S., Robbins K.A. (2013). Detecting alpha spindle events in EEG time series using adaptive autoregressive models. BMC Neurosci..

[108] Simon M., Schmidt E.A., Kincses W.E., Fritzsche M., Bruns A., Aufmuth C., Bogdan M., Rosenstiel W., Schrauf M. (2011). EEG alpha spindle measures as indicators of driver fatigue under real traffic conditions. Clin. Neurophysiol..

[109] Mu Z., Hu J., Min J. (2017). Driver Fatigue Detection System Using Electroencephalography Signals Based on Combined Entropy Features. Appl. Sci..

[110] Zhang Z., Luo D., Rasim Y., Li Y., Meng G., Xu J., Wang C. (2016). A Vehicle Active Safety Model: Vehicle Speed Control Based on Driver Vigilance Detection Using Wearable EEG and Sparse Representation. Sensors.

[111] Caffier P.P., Erdmann U., Ullsperger P. (2003). Experimental evaluation of eye-blink parameters as a drowsiness measure. Eur. J. Appl. Physiol..

[112] Johns M.W., Tucker A., Chapman R., Crowley K., Michael N. (2007). Monitoring eye and eyelid movements by infrared reflectance oculography to measure drowsiness in drivers. Somnologie Schlafforschung Und Schlafmed..

[113] Jia-Xin M., Li-Chen S., Bao-Liang L. (2014). An EOG-based Vigilance Estimation Method Applied for Driver Fatigue Detection. Neurosci. Biomed. Eng. (Discontin.).

[114] Magosso E., Provini F., Montagna P., Ursino M. (2006). A wavelet based method for automatic detection of slow eye movements: A pilot study. Med. Eng. Phys..

[115] Miles W.R. (1929). Horizontal eye movements at the onset of sleep. Psychol. Rev..

[116] Freitas I. (2008). Fatigue Detection in EMG Signals. Master’s Thesis.

[117] Picot A., Charbonnier S., Caplier A. (2011). EOG-based drowsiness detection: Comparison between a fuzzy system and two supervised learning classifiers. IFAC Proc. Vol..

[118] De Gennaro L., Ferrara M., Bertini M. (2001). The boundary between wakefulness and sleep: Quantitative electroencephalographic changes during the sleep onset period. Neuroscience.

[119] Zheng Y., Li S.E., Wang J., Cao D., Li K. (2016). Stability and Scalability of Homogeneous Vehicular Platoon: Study on the Influence of Information Flow Topologies. IEEE Trans. Intell. Transp. Syst..

[120] Wang F.-Y. (2010). Parallel control and management for intelligent transportation systems: Concepts, architectures, and applications. IEEE Trans. Intell. Transp. Syst..

[121] Li G., Li S.E., Cheng B., Green P. (2017). Estimation of driving style in naturalistic highway traffic using maneuver transition probabilities. Transp. Res. Part C Emerg. Technol..

[122] Wang W., Xi J., Chen H. (2014). Modeling and recognizing driver behavior based on driving data: A survey. Math. Probl. Eng..

[123] Fazeen M., Gozick B., Dantu R., Bhukhiya M., González M.C. (2012). Safe Driving Using Mobile Phones. Ieee Trans. Intell. Transp. Syst..

[124] Li G., Li S.E., Cheng B. (2015). Field operational test of advanced driver assistance systems in typical Chinese road conditions: The influence of driver gender, age and aggression. Int. J. Automot. Technol..

[125] Filev D., Lu J., Prakah-Asante K., Tseng F. Real-time driving behavior identification based on driver-in-the-loop vehicle dynamics and control. Proceedings of the IEEE International Conference on Systems, Man and Cybernetics.

[126] Lee T., Son J. (2011). Relationships between Driving Style and Fuel Consumption in Highway Driving.

[127] Rajan B.V.P., McGordon A., Jennings P.A. (2012). An Investigation on the Effect of Driver Style and Driving Events on Energy Demand of a PHEV. World Electr. Veh. J..

[128] Johnson D.A., Trivedi M.M. Driving style recognition using a smartphone as a sensor platform. Proceedings of the 2011 14th International IEEE Conference on Intelligent Transportation Systems (ITSC).

[129] Syed F., Nallapa S., Dobryden A., Grand C., McGee R., Filev D. (2010). Design and Analysis of an Adaptive Real-Time Advisory System for Improving Real World Fuel Economy in a Hybrid Electric Vehicle.

[130] Karginova N., Byttner S., Svensson M. (2012). Data-Driven Methods for Classification of Driving Styles in Buses.

[131] Vaitkus V., Lengvenis P., Žylius G. Driving style classification using long-term accelerometer information. Proceedings of the 19th International Conference on Methods and Models in Automation and Robotics (MMAR).

[132] Jachimczyk B., Dziak D., Czapla J., Damps P., Kulesza W.J. (2018). IoT On-Board System for Driving Style Assessment. Sensors.

[133] Wang R., Lukic S.M. Review of driving conditions prediction and driving style recognition based control algorithms for hybrid electric vehicles. Proceedings of the IEEE Vehicle Power and Propulsion Conference.

[134] Dörr D., Grabengiesser D., Gauterin F. Online driving style recognition using fuzzy logic. Proceedings of the 17th International IEEE Conference on Intelligent Transportation Systems (ITSC).

[135] Xu L., Hu J., Jiang H., Meng W. (2015). Establishing Style-Oriented Driver Models by Imitating Human Driving Behaviors. IEEE Trans. Intell. Transp. Syst..

[136] Murphey Y.L., Milton R., Kiliaris L. Driver’s style classification using jerk analysis. Proceedings of the IEEE Workshop on Computational Intelligence in Vehicles and Vehicular Systems.

[137] Gilman E., Keskinarkaus A., Tamminen S., Pirttikangas S., Röning J., Riekki J. (2015). Personalised assistance for fuel-efficient driving. Transp. Res. Part C Emerg. Technol..

[138] Sysoev M., Kos A., Guna J., Pogačnik M. (2017). Estimation of the Driving Style Based on the Users’ Activity and Environment Influence. Sensors.

[139] Constantinescu Z., Marinoiu C., Vladoiu M. (2010). Driving style analysis using data mining techniques. Int. J. Comput. Commun. Control.

[140] Taubman-Ben-Ari O., Mikulincer M., Gillath O. (2004). The multidimensional driving style inventory—Scale construct and validation. Accid. Anal. Prev..

[141] Manzoni V., Corti A., Luca P.D., Savaresi S.M. Driving style estimation via inertial measurements. Proceedings of the 13th International IEEE Conference on Intelligent Transportation Systems.

[142] Neubauer J.S., Wood E. (2013). Accounting for the Variation of Driver Aggression in the Simulation of Conventional and Advanced Vehicles.

[143] Corti A., Ongini C., Tanelli M., Savaresi S.M. Quantitative Driving Style Estimation for Energy-Oriented Applications in Road Vehicles. Proceedings of the 2013 IEEE International Conference on Systems, Man, and Cybernetics.

[144] Augustynowicz A. (2009). Preliminary classification of driving style with objective rank method. Int. J. Automot. Technol..

[145] Ericsson E. (2001). Independent driving pattern factors and their influence on fuel-use and exhaust emission factors. Transp. Res. Part D Transp. Environ..

[146] Syed F.U., Filev D., Ying H. Fuzzy Rule-Based Driver Advisory System for Fuel Economy Improvement in a Hybrid Electric Vehicle. Proceedings of the NAFIPS 2007—2007 Annual Meeting of the North American Fuzzy Information Processing Society.

[147] Langari R., Jong-Seob W. (2005). Intelligent energy management agent for a parallel hybrid vehicle-part I: System architecture and design of the driving situation identification process. IEEE Trans. Veh. Technol..

[148] Kim J., Sim H., Oh J. (2012). The Flexible EV/HEV and SOC Band Control Corresponding to Driving Mode, Driver’s Driving Style and Environmental Circumstances.

[149] Stoichkov R. (2013). Android Smartphone Application for Driving Style Recognition.

[150] Miyajima C., Nishiwaki Y., Ozawa K., Wakita T., Itou K., Takeda K., Itakura F. (2007). Driver Modeling Based on Driving Behavior and Its Evaluation in Driver Identification. Proc. IEEE.

[151] Mudgal A., Hallmark S., Carriquiry A., Gkritza K. (2014). Driving behavior at a roundabout: A hierarchical Bayesian regression analysis. Transp. Res. Part D Transp. Environ..

[152] Ly M.V., Martin S., Trivedi M.M. Driver classification and driving style recognition using inertial sensors. Proceedings of the IEEE Intelligent Vehicles Symposium (IV).

[153] Mizumachi M., Kaminuma A., Ono N., Ando S. (2014). Robust Sensing of Approaching Vehicles Relying on Acoustic Cues. Sensors.

[154] Kim J., Hong S., Baek J., Kim E., Lee H. Autonomous vehicle detection system using visible and infrared camera. Proceedings of the 2012 12th International Conference on Control, Automation and Systems.

[155] Wang C.R., Lien J.J. (2008). Automatic Vehicle Detection Using Local Features—A Statistical Approach. IEEE Trans. Intell. Transp. Syst..

[156] Sen R., Siriah P., Raman B. RoadSoundSense: Acoustic sensing based road congestion monitoring in developing regions. Proceedings of the 8th Annual IEEE Communications Society Conference on Sensor, Mesh and Ad Hoc Communications and Networks.

[157] Van Leeuwen M.B., Groen F.C.A. (2005). Vehicle detection with a mobile camera: Spotting midrange, distant, and passing cars. IEEE Robot. Autom. Mag..

[158] Ieng S., Vrignon J., Gruyer D., Aubert D. A new multi-lanes detection using multi-camera for robust vehicle location. Proceedings of the IEEE Intelligent Vehicles Symposium.

[159] For W.-K., Leman K., Eng H.-L., Chew B.-F., Wan K.-W. A multi-camera collaboration framework for real-time vehicle detection and license plate recognition on highways. Proceedings of the IEEE Intelligent Vehicles Symposium.

[160] Hirvonen P.C.D., Southall T.C.B. Stereo-Based Object Detection, Classification, and Quantitative Evaluation with Automotive Applications. Proceedings of the IEEE Computer Society Conference on Computer Vision and Pattern Recognition (CVPR’05)-Workshops.

[161] Lim Y.-C., Lee C.-H., Kwon S., Kim J. Event-driven track management method for robust multi-vehicle tracking. Proceedings of the IEEE Intelligent Vehicles Symposium (IV).

[162] Kowsari T., Beauchemin S.S., Cho J. Real-time vehicle detection and tracking using stereo vision and multi-view AdaBoost. Proceedings of the 14th International IEEE Conference on Intelligent Transportation Systems (ITSC).

[163] Cho H.-J., Tseng M.-T. (2013). A support vector machine approach to CMOS-based radar signal processing for vehicle classification and speed estimation. Math. Comput. Model..

[164] Nashashibi F., Bargeton A. Laser-based vehicles tracking and classification using occlusion reasoning and confidence estimation. Proceedings of the IEEE Intelligent Vehicles Symposium.

[165] Blanc C., Aufrere R., Malaterre L., Gallice J., Alizon J. (2004). Obstacle detection and tracking by millimeter wave radar. IFAC Proc. Vol..

[166] Sun X., Zhou Z., Zhao C., Zou W. A compressed sensing radar detection scheme for closing vehicle detection. Proceedings of the 2012 IEEE International Conference on Communications (ICC).

[167] Sang Jin P., Tae Yong K., Sung Min K., Kyung Heon K. A novel signal processing technique for vehicle detection radar. Proceedings of the IEEE MTT-S International Microwave Symposium Digest.

[168] Biondi F., Rossi R., Gastaldi M., Mulatti C. (2014). Beeping ADAS: Reflexive effect on drivers’ behavior. Transp. Res. Part F Traffic Psychol. Behav..

[169] Natale D.J., Tutwiler R.L., Baran M.S., Durkin J.R. Using full motion 3D Flash LIDAR video for target detection, segmentation, and tracking. Proceedings of the IEEE Southwest Symposium on Image Analysis & Interpretation (SSIAI).

[170] Zeng Y., Yu H., Dai H., Song S., Lin M., Sun B., Jiang W., Meng M.Q.-H. (2018). An Improved Calibration Method for a Rotating 2D LIDAR System. Sensors.

[171] Kadow U., Schneider G., Vukotich A. Radar-Vision Based Vehicle Recognition with Evolutionary Optimized and Boosted Features. Proceedings of the 2007 IEEE Intelligent Vehicles Symposium.

[172] Velazquez-Pupo R., Sierra-Romero A., Torres-Roman D., Shkvarko Y.V., Santiago-Paz J., Gómez-Gutiérrez D., Robles-Valdez D., Hermosillo-Reynoso F., Romero-Delgado M. (2018). Vehicle Detection with Occlusion Handling, Tracking, and OC-SVM Classification: A High Performance Vision-Based System. Sensors.

[173] Ling B., Gibson D.R.P., Middleton D. (2013). Motorcycle Detection and Counting Using Stereo Camera, IR Camera, and Microphone Array.

[174] Blanc C., Trassoudaine L., Gallice J. EKF and particle filter track-to-track fusion: A quantitative comparison from radar/lidar obstacle tracks. Proceedings of the 7th International Conference on Information Fusion.

[175] Chellappa R., Gang Q., Qinfen Z. Vehicle detection and tracking using acoustic and video sensors. Proceedings of the IEEE International Conference on Acoustics, Speech, and Signal Processing.

[176] Stiller C., Hipp J., Rössig C., Ewald A. (2000). Multisensor obstacle detection and tracking. Image Vis. Comput..

[177] F S.A.R., Frémont V., Bonnifait P., Cherfaoui V. Visual confirmation of mobile objects tracked by a multi-layer lidar. Proceedings of the 13th International IEEE Conference on Intelligent Transportation Systems.

[178] Premebida C., Monteiro G., Nunes U., Peixoto P. A Lidar and Vision-based Approach for Pedestrian and Vehicle Detection and Tracking. Proceedings of the IEEE Intelligent Transportation Systems Conference.

[179] Fritsch J., Michalke T., Gepperth A., Bone S., Waibel F., Kleinehagenbrock M., Gayko J., Goerick C. Towards a human-like vision system for Driver Assistance. Proceedings of the IEEE Intelligent Vehicles Symposium.

[180] Tan Y., Han F., Ibrahim F. A Radar Guided Vision System for Vehicle Validation and Vehicle Motion Characterization. Proceedings of the 2007 IEEE Intelligent Transportation Systems Conference.

[181] Alefs B., Schreiber D., Clabian M. Hypothesis based vehicle detection for increased simplicity in multi-sensor ACC. Proceedings of the IEEE Intelligent Vehicles Symposium.

[182] Feng L., Sparbert J., Stiller C. IMMPDA vehicle tracking system using asynchronous sensor fusion of radar and vision. Proceedings of the IEEE Intelligent Vehicles Symposium.

[183] Bertozzi M., Bombini L., Cerri P., Medici P., Antonello P.C., Miglietta M. Obstacle detection and classification fusing radar and vision. Proceedings of the IEEE Intelligent Vehicles Symposium.

[184] Alessandretti G., Broggi A., Cerri P. (2007). Vehicle and Guard Rail Detection Using Radar and Vision Data Fusion. IEEE Trans. Intell. Transp. Syst..

[185] Garcia F., Cerri P., Broggi A., Escalera A.d.l., Armingol J.M. Data fusion for overtaking vehicle detection based on radar and optical flow. Proceedings of the 2012 IEEE Intelligent Vehicles Symposium.

[186] Kmiotek P., Kmiotek P., Ruichek Y. Multisensor fusion based tracking of coalescing objects in urban environment for an autonomous vehicle navigation. Proceedings of the IEEE International Conference on Multisensor Fusion and Integration for Intelligent Systems.

[187] Bakr M.A., Lee S. (2017). Distributed Multisensor Data Fusion under Unknown Correlation and Data Inconsistency. Sensors.

[188] Audi|Luxury Sedans, SUVs, Convertibles, Electric Vehicles & More. https://www.audiusa.com/.

[189] Bmw The international BMW Website|BMW.com. https://www.bmw.com/en/index.html.

[190] New Cars, Trucks, SUVs & Hybrids|Toyota Official Site. https://www.toyota.com.

[191] Mercedes-Benz International: News, Pictures, Videos & Livestreams. https://www.mercedes-benz.com/content/com/en.

[192] Ford Ford—New Cars, Trucks, SUVs, Crossovers & Hybrids|Vehicles Built Just for You|Ford.com. https://www.ford.com/.

[193] Kurihata H., Takahashi T., Ide I., Mekada Y., Murase H., Tamatsu Y., Miyahara T. Rainy weather recognition from in-vehicle camera images for driver assistance. Proceedings of the IEEE Intelligent Vehicles Symposium.

[194] Li L., Jingyan S., Fei-Yue W., Wolfgang N., Nan-Ning Z. (2005). IVS 05: New developments and research trends for intelligent vehicles. IEEE Intell. Syst..

[195] Lee J., Hoffman J., Stoner H., Seppelt B., Brown M. Application of ecological interface design to driver support systems. Proceedings of the IEA 16th World Congress on Ergonomics.

[196] Horter M.H., Stiller C., Koelen C. A hardware and software framework for automotive intelligent lighting. Proceedings of the IEEE Intelligent Vehicles Symposium.

[197] Halimeh J.C., Roser M. Raindrop detection on car windshields using geometric-photometric environment construction and intensity-based correlation. Proceedings of the IEEE Intelligent Vehicles Symposium.

[198] Gormer S., Kummert A., Park S., Egbert P. Vision-based rain sensing with an in-vehicle camera. Proceedings of the 2009 IEEE Intelligent Vehicles Symposium.

[199] Li Z., Li L., Zhang Y. (2009). IVS 09: Future Research in Vehicle Vision Systems. IEEE Intell. Syst..

[200] Rubio J.C., Serrat J., López A.M., Ponsa D. Multiple target tracking for intelligent headlights control. Proceedings of the 13th International IEEE Conference on Intelligent Transportation Systems.

[201] David K., Flach A. (2010). CAR-2-X and Pedestrian Safety. IEEE Veh. Technol. Mag..

[202] Sivak M. (1996). The information that drivers use: Is it indeed 90% visual?. Perception.

[203] Hahn W. Vision enhancement-concepts for the future?. Proceedings of the International Technical Conference on the Enhanced Safety of Vehicles.

[204] Lim J.H., Tsimhoni O., Liu Y. (2010). Investigation of Driver Performance with Night Vision and Pedestrian Detection Systems—Part I: Empirical Study on Visual Clutter and Glance Behavior. IEEE Trans. Intell. Transp. Syst..

[205] Vicente K.J., Rasmussen J. (1992). Ecological interface design: Theoretical foundations. IEEE Trans. Syst. Man Cybern..

[206] Kaber D.B., Riley J.M., Tan K.-W., Endsley M.R. (2001). On the Design of Adaptive Automation for Complex Systems. Int. J. Cogn. Ergon..

[207] Stanton N.A., Hedge A., Brookhuis K., Salas E., Hendrick H.W. (2004). Handbook of Human Factors and Ergonomics Methods.

[208] Damiani S., Deregibus E., Andreone L. (2009). Driver-vehicle interfaces and interaction: Where are they going?. Eur. Transp. Res. Rev..

[209] Salvucci D.D. Predicting the effects of in-car interfaces on driver behavior using a cognitive architecture. Proceedings of the SIGCHI Conference on Human Factors in Computing Systems.

[210] Rothrock L., Koubek R., Fuchs F., Haas M., Salvendy G. (2002). Review and reappraisal of adaptive interfaces: Toward biologically inspired paradigms. Theor. Issues Ergon. Sci..

[211] Burnett G. A road-based evaluation of different positions for an in-vehicle navigation display. Proceedings of the International Conference of Traffic and Transport Psychology.

[212] Bradley D.P. (2004). The TIMIDDS Project: Transparent Integration of Multiple Intelligent Data and Display Systems. Bachelor Thesis.

[213] Champoux B. A mode of interaction for driver vehicle interface (DVI). Proceedings of the IEEE Intelligent Vehicles Symposium.

[214] Jahn G., Oehme A., Krems J.F., Gelau C. (2005). Peripheral detection as a workload measure in driving: Effects of traffic complexity and route guidance system use in a driving study. Transp. Res. Part F Traffic Psychol. Behav..

[215] Caleefato C., Montanari R., Tango F. (2007). Advanced Drivers Assistant Systems in Automation.

[216] Scott J.J., Gray R. (2008). A Comparison of Tactile, Visual, and Auditory Warnings for Rear-End Collision Prevention in Simulated Driving. Hum. Factors.

[217] Kim S., Sekiyama K., Fukuda T. User-Adaptive Interface with Reconfigurable Keypad for In-vehicle Information Systems. Proceedings of the International Symposium on Micro-Nano Mechatronics and Human Science.

[218] Niedermaier B., Durach S., Eckstein L., Keinath A. (2009). The New BMW iDrive—Applied Processes and Methods to Assure High Usability.

[219] Hofmann P., Rinkenauer G., Gude D. (2010). Preparing lane changes while driving in a fixed-base simulator: Effects of advance information about direction and amplitude on reaction time and steering kinematics. Transp. Res. Part F Traffic Psychol. Behav..

[220] Amditis A., Andreone L., Pagle K., Markkula G., Deregibus E., Rue M.R., Bellotti F., Engelsberg A., Brouwer R., Peters B. (2010). Towards the Automotive HMI of the Future: Overview of the AIDE-Integrated Project Results. IEEE Trans. Intell. Transp. Syst..

[221] Hollnagel E. (2006). A function-centred approach to joint driver-vehicle system design. Cogn. Technol. Work.

[222] Amberkar S., Bolourchi F., Demerly J., Millsap S. (2004). A Control System Methodology for Steer by Wire Systems.

[223] Yih P. (2005). Steer-by-Wire: Implications for Vehicle Handling and Safety. Ph.D. Thesis.

[224] Haggag S., Rosa A., Huang K., Cetinkunt S. (2007). Fault tolerant real time control system for steer-by-wire electro-hydraulic systems. Mechatronics.

[225] Chen L.-K., Shieh B.-J. (2008). Coordination of the authority between the vehicle driver and a steering assist controller. WSEAS Trans. Syst. Control.

[226] Hayama R., Kawahara S., Nakano S., Kumamoto H. (2010). Resistance torque control for steer-by-wire system to improve human–machine interface. Veh. Syst. Dyn..

[227] Petersen A., Barrett R., Morrison S. (2006). Driver-training and emergency brake performance in cars with antilock braking systems. Saf. Sci..

[228] Mulder M., Mulder M., van Paassen M.M., Abbink D.A. (2008). Haptic gas pedal feedback. Ergonomics.

[229] Wada T., Doi S., Tsuru N., Isaji K., Kaneko H. (2010). Characterization of Expert Drivers’ Last-Second Braking and Its Application to a Collision Avoidance System. IEEE Trans. Intell. Transp. Syst..

[230] Lee S.-D., Kim S.-L. (2010). Characterization and Development of the Ideal Pedal Force, Pedal Travel, and Response Time in the Brake System for the Translation of the Voice of the Customer to Engineering Specifications. Proc. Inst. Mech. Eng. Part D J. Automob. Eng..

[231] Amditis A., Bimpas M., Thomaidis G., Tsogas M., Netto M., Mammar S., Beutner A., Mohler N., Wirthgen T., Zipser S. (2010). A Situation-Adaptive Lane-Keeping Support System: Overview of the SAFELANE Approach. IEEE Trans. Intell. Transp. Syst..

[232] Glaser S., Vanholme B., Mammar S., Gruyer D., Nouveliere L. (2010). Maneuver-Based Trajectory Planning for Highly Autonomous Vehicles on Real Road with Traffic and Driver Interaction. IEEE Trans. Intell. Transp. Syst..

[233] Plöchl M., Edelmann J. (2007). Driver models in automobile dynamics application. Veh. Syst. Dyn..

[234] Yih P., Ryu J., Gerdes J.C. Modification of vehicle handling characteristics via steer-by-wire. Proceedings of the 2003 American Control Conference.

[235] Hoedemaeker M., Brookhuis K.A. (1998). Behavioural adaptation to driving with an adaptive cruise control (ACC). Transp. Res. Part F Traffic Psychol. Behav..

[236] Inagaki T. (2008). Smart collaboration between humans and machines based on mutual understanding. Annu. Rev. Control.

[237] Walker G.H., Stanton N.A., Young M.S. (2006). The ironies of vehicle feedback in car design. Ergonomics.

[238] Jones W.D. (2001). Keeping cars from crashing. IEEE Spectr..

[239] Jones W.D. (2002). Building safer cars. IEEE Spectr..

[240] Inagaki T., Furukawa H., Itoh M. Human interaction with adaptive automation: Strategies for trading of control under possibility of over-trust and complacency. Proceedings of the HCI International 2005.

[241] Pauwelussen J., Feenstra P.J. (2010). Driver Behavior Analysis during ACC Activation and Deactivation in a Real Traffic Environment. IEEE Trans. Intell. Transp. Syst..

[242] Adell E., Várhelyi A., Fontana M.d. (2011). The effects of a driver assistance system for safe speed and safe distance—A real-life field study. Transp. Res. Part C Emerg. Technol..

[243] Duan J., Li R., Hou L., Wang W., Li G., Li S.E., Cheng B., Gao H. (2017). Driver braking behavior analysis to improve autonomous emergency braking systems in typical Chinese vehicle-bicycle conflicts. Accid. Anal. Prev..

[244] Noy I.Y., Shinar D., Horrey W.J. (2018). Automated driving: Safety blind spots. Saf. Sci..

[245] Chang N., Pan S., Srinivasan K., Feng Z., Xia W., Pawlak T., Geb D. (2018). Emerging ADAS Thermal Reliability Needs and Solutions. IEEE Micro.

[246] Shin D., Kim B., Yi K., Carvalho A., Borrelli F. (2019). Human-Centered Risk Assessment of an Automated Vehicle Using Vehicular Wireless Communication. IEEE Trans. Intell. Transp. Syst..

[247] Sawade O., Schulze M., Radusch I. (2018). Robust Communication for Cooperative Driving Maneuvers. IEEE Intell. Transp. Syst. Mag..

[248] ISO 26262-1:2018. http://www.iso.org/cms/render/live/en/sites/isoorg/contents/data/standard/06/83/68383.html.

[249] Palin R., Ward D., Habli I., Rivett R. ISO 26262 safety cases: Compliance and assurance. Proceedings of the 6th IET International Conference on System Safety.

